# Density structure of Earth's lowermost mantle from Stoneley mode splitting observations

**DOI:** 10.1038/ncomms15241

**Published:** 2017-05-15

**Authors:** Paula Koelemeijer, Arwen Deuss, Jeroen Ritsema

**Affiliations:** 1 Department of Earth Sciences, ETH Zürich, 8092 Zürich, Switzerland; 2 Department of Earth Sciences, University of Oxford, OX1 3AN Oxford, UK; 3 Department of Earth Sciences, Utrecht University, 3508 TC Utrecht, The Netherlands; 4 Department of Earth and Environmental Sciences, University of Michigan, Ann Arbor, Michigan 48109-1005, USA

## Abstract

Advances in our understanding of Earth's thermal evolution and the style of mantle convection rely on robust seismological constraints on lateral variations of density. The large-low-shear-wave velocity provinces (LLSVPs) atop the core–mantle boundary beneath Africa and the Pacific are the largest structures in the lower mantle, and hence severely affect the convective flow. Here, we show that anomalous splitting of Stoneley modes, a unique class of free oscillations that are perturbed primarily by velocity and density variations at the core–mantle boundary, is explained best when the overall density of the LLSVPs is lower than the surrounding mantle. The resolved density variations can be explained by the presence of post-perovskite, chemical heterogeneity or a combination of the two. Although we cannot rule out the presence of a ∼100-km-thick denser-than-average basal structure, our results support the hypothesis that LLSVPs signify large-scale mantle upwelling in two antipodal regions of the mantle.

## Abstract (web summary)

Large-low-shear-wave velocity provinces (LLSVPs) are present at the core–mantle boundary. Here, the authors show that splitting of Stoneley modes is probably due to a lower density of LLSVPs than the surrounding mantle because of the presence of post-perovskite, chemical heterogeneity or both.

The two large-low-shear-wave velocity provinces (LLSVPs) in the lower mantle beneath Africa and the Pacific stand out in every global-scale seismic map of the Earth's lower mantle (Fig. 1)^1^,^2^,^3^. The LLSVPs cover about a quarter of the core–mantle boundary (CMB) and lower-than-average shear-wave velocities are observed more than 1,000 km up into the mantle. While defined by their low shear-wave velocities, they are also characterized by sharp margins^4^ and high ratios of shear- to compressional-wave velocity variations^5^,^6^. These structures have been interpreted as long-lived, chemically distinct piles^7^,^8^,^9^, as broad thermal upwellings^10^,^11^,^12^, and as clusters of narrow plumes^13^.

To test models of the origin, composition, and longevity of LLSVPs, it is crucial to constrain the density structure in the mantle using observations of the splitting of normal modes (that is, whole-Earth seismic oscillations). Accurate estimates of density variations are essential for modelling mantle flow and to distinguish between a thermal or compositional origin of mantle heterogeneity. Some previous inversions^14^ and statistical analyses^15^,^16^ of spheroidal normal-mode splitting data have suggested that the density of the LLSVPs is relatively high, which cannot be reconciled with a purely thermal origin^17^. However, these studies relied primarily on observations of the splitting of modes with frequencies below 3 mHz (refs 18, 19) and with a sensitivity to both the upper and lower mantle. It has been demonstrated that such modes have insufficient resolving power to constrain the sign of the density variations in the LLSVPs^20^,^21^,^22^.

Here, we analyse a new collection of spheroidal normal mode splitting measurements that is based on new Global Seismic Network recordings of mega-thrust and strong continental earthquakes from the past decades. Our data set is extended to 10 mHz (ref. 23), and includes for the first time, measurements of CMB Stoneley modes (from hereon simply called Stoneley modes)^24^. Stoneley modes oscillate primarily in the lowermost mantle and the outermost core. Therefore, they are sensitive only to the velocity and density structure near the CMB, as illustrated by modes _2_*S*_16_ and _3_*S*_26_ (Fig. 2a,f). These, and all other, Stoneley modes exhibit the characteristic degree-2 pattern of seismic heterogeneity in the lowermost mantle (Fig. 2b,g), similar to the pattern observed in diffracted wave travel times^24^. The pattern of the splitting functions is reproduced well by mantle model SP12RTS with correlation coefficients higher than 0.90 (Fig. 2c,h).

To explicitly test whether the density in the LLSVPs is higher or lower than the ambient mantle, we analyse the new mode measurements following a straightforward model space search with few free parameters. Using a robust statistical measure, we determine the probability that the splitting function measurements are optimally fit within their uncertainties by different density models. For reasonable estimates of the velocity structure, we consistently find that Stoneley mode splitting functions are fitted best by overall low-density LLSVPs. We conclude therefore that the LLSVPs signify large-scale positively buoyant regions in the Earth's mantle, consistent with other geophysical observables^25^,^26^. The resolved density variations cannot be uniquely interpreted in terms of purely thermal or thermochemical structures in the Earth's deep mantle, nor can we rule out that the LLSVPs are dense at their very base (<100 km).

## Results

### Splitting function predictions

**Figure 1:**
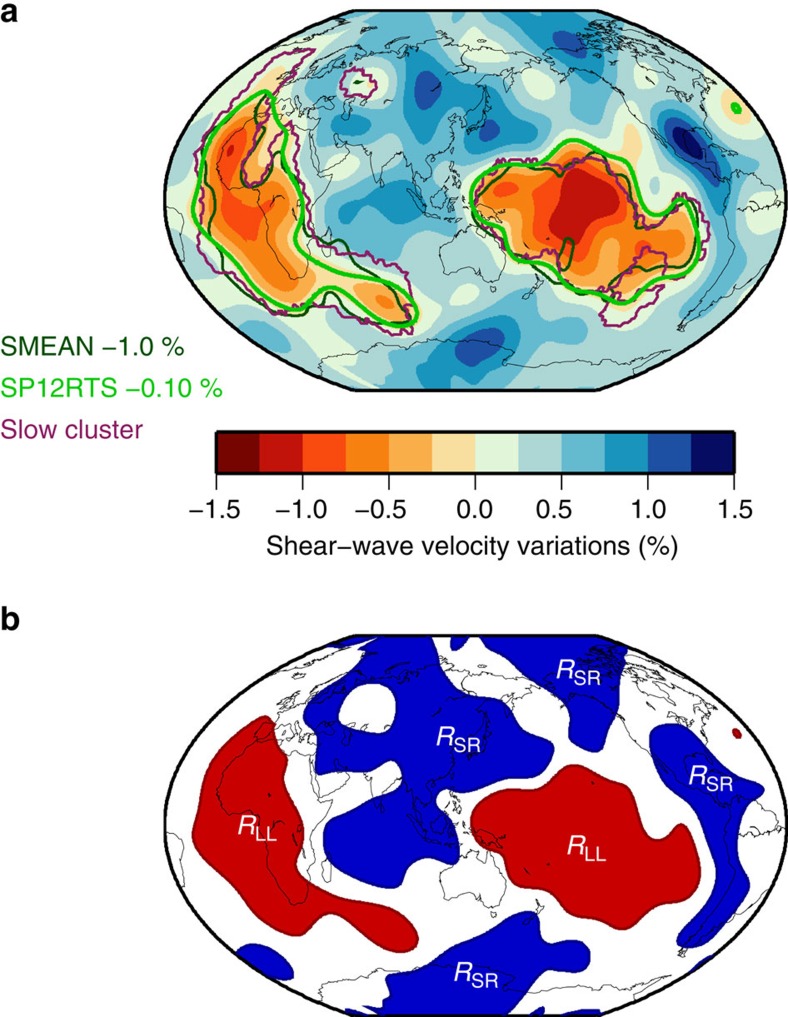
Extent of the LLSVPs in the lowermost mantle. (**a**) The extent of the LLSVPs (‘LL' region) is indicated for the -0.10% velocity contour of SP12RTS (ref. 27), the −1.0% velocity contour of SMEAN^66^ and the slow lower mantle cluster^2^, drawn over the shear-wave velocity structure of SP12RTS at 2,850 km depth. All definitions result in a similar lateral extent. (**b**) Schematic overview of the ‘LL' (red) and ‘SR' (blue) regions in the lowermost mantle used in this study. Density structure is described by scaling factors *R*_LL_ and *R*_SR_ in the two regions. A scaling of *R*=0.3 is used in the remaining areas (white), resulting in small density variations of <0.15%.

Model SP12RTS (ref. 27; Supplementary Fig. 1) is used to describe the independently constrained shear-wave (dln*V*_S_) and compressional-wave (dln*V*_P_) velocity structure throughout the mantle. SP12RTS assumes a scaling factor to describe density variations dln*ρ* of *R*=dln*ρ*/dln*V*_S_=0.3, as expected for purely thermal variations^17^. In our modelling, we assume that above 2,500 km depth, the density and shear-wave velocity variations are perfectly correlated and scaled by *R*=0.3, consistent with SP12RTS. Below 2,500 km depth, the scaling factor *R*_LL_ for the LLSVPs (defined by dln*V*_S_<−0.10%) and the scaling factor *R*_SR_ for the regions surrounding the LLSVPs (defined by dln*V*_S_>0.50%) are free parameters (Fig. 1b and Supplementary Fig. 2). As lateral variations in CMB topography also perturb the splitting of Stoneley modes^28^ (Supplementary Fig. 3), we incorporate CMB topography as a third model parameter *H*, which scales CMB topography variations to lower mantle density variations (see Methods).

Normal mode splitting function predictions are used to determine the probability of each density input model for every combination of *R*_LL_, *R*_SR_ and *H*. We define the model probability for a particular splitting function as a conditional sum of inverse uncertainties for those coefficients that are fit within their uncertainties, normalized by the sum of all inverse uncertainties (see Methods). By using this measure of fit, we consider all models that fit the measurements equally within the given uncertainties and we ensure that most emphasis is given to measured coefficients with the smallest uncertainties. A similar definition of likelihood has been applied to demonstrate the presence of post-perovskite at the CMB using core-diffracted wave data^29^. More detailed information on SP12RTS and the density input models can be found in the Methods section.

**Figure 2:**
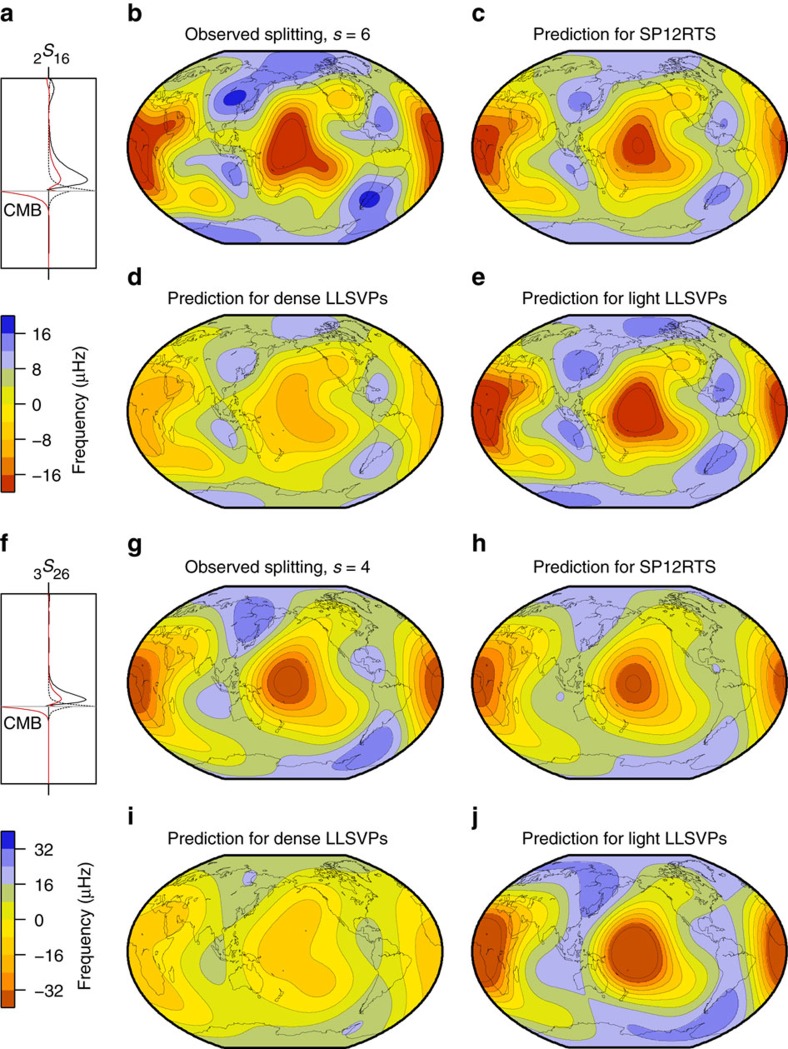
Observed and predicted Stoneley mode splitting function maps. (**a**,**f**) Sensitivity kernels for density (red), shear-wave velocity (solid) and compressional-wave velocity (dashed) structure for modes _2_*S*_16_ and _3_*S*_26_, respectively. The radius of the CMB is indicated by a horizontal line. (**b**) Observed splitting for _2_*S*_16_ plotted up to maximum structural degree *s*=6. (**c**) Predicted splitting for mantle model SP12RTS (ref. 27). (**d**) Predicted splitting for dense LLSVPs (*R*_LL_=−4 and *R*_SR_=+0.3). (**e**) Predicted splitting for light LLSVPs (*R*_LL_=+4 and *R*_SR_=+0.3). (**g**–**j**) Similar as (**b**–**e**) but for mode _3_*S*_26_ up to *s*=4. CMB topography variations are excluded.

The default model of SP12RTS with *R*_LL_=*R*_SR_=+0.3 and *H*=0 underestimates the splitting function amplitudes (see Supplementary Note 1 for a discussion on the robustness of these) by a factor of 0.75–0.9 (Fig. 2c,h). If we assume *R*_LL_<0 (and *R*_SR_=0.3 with *H*=0), as suggested in previous studies, the splitting function amplitudes are underestimated even more (Fig. 2d,i). We match the amplitudes of the splitting functions for _3_*S*_26_ and _2_*S*_16_ only when *R*_LL_ is positive if *H*=0 (Fig. 2e,j). SP12RTS also underpredicts the amplitudes of other Stoneley mode splitting functions and again their amplitudes are matched better for models with *R*_LL_>0 (Supplementary Fig. 4 and Supplementary Note 2). When *H*≠0, we observe the expected trade-off between LLSVP density and CMB topography in the predicted splitting functions (Supplementary Fig. 5 and Supplementary Note 3), as further discussed below.

### Model space search

**Figure 3:**
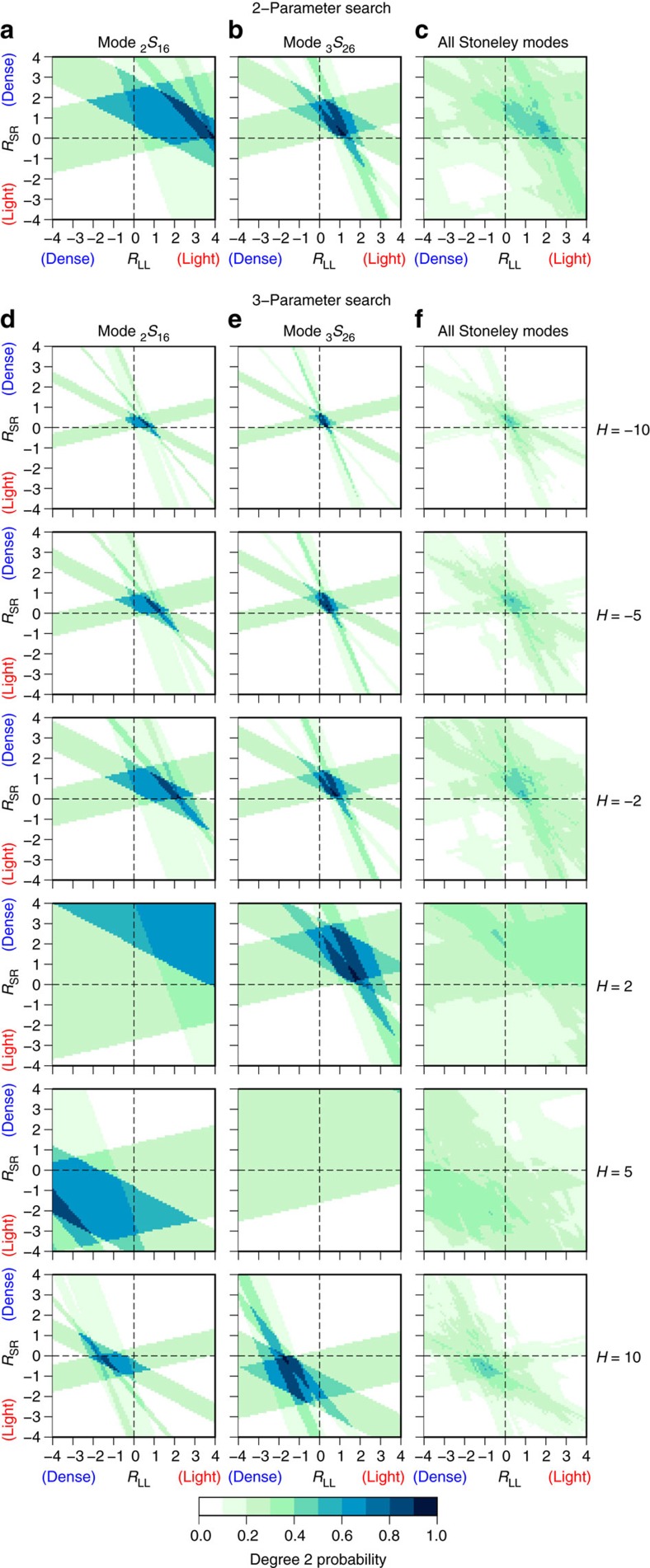
Probability of density models for individual modes and mode selections. (**a**–**c**) Two-parameter search without CMB topography variations for **a** Stoneley mode _2_*S*_16_, **b** Stoneley mode _3_*S*_26_ and **c** all Stoneley modes together. Darker colours indicate a better fit to the measurements and areas with the same colour fit equally well given the data uncertainties. (**d**–**f**) Similar as (**a**–**c**) but for the three-parameter search including CMB topography variations. The probability of density models is shown for different values of *H*, the scaling factor between lower mantle density variations and CMB topography, as indicated on the right of each row. Negative values of *H* correspond to dynamically feasible models.

We focus on structural degree *s*=2, which is the dominant and best determined spherical harmonic degree of heterogeneity in the lowermost mantle. Firstly, we consider the results of the simpler, two-parameter search of only *R*_LL_ and *R*_SR_ (with *H*=0) before we discuss the results of the three-parameter search, where we include CMB topography variations. Figure 3a–c indicate that it is most likely (as quantified by the probabilities) that, when *H*=0, the splitting functions of Stoneley modes are reproduced within uncertainty when *R*_LL_ and *R*_SR_ are both positive. This means that the Stoneley mode data prefer relatively light LLSVPs surrounded by dense regions, compared to the radial average. For Stoneley mode _2_*S*_16_ (Fig. 3a), density models with values *R*_LL_=+3.6 to +4.0 and *R*_SR_=+0.1 to+0.6 have the highest probability of 1.0. The splitting function for _3_*S*_26_, which has a more focused sensitivity to the lowermost mantle (Fig. 2f), is optimally fit (probability of 1.0) for smaller scaling factors: *R*_LL_=+0.9 to +1.3 and *R*_SR_=+0.2 to +0.6 (Fig. 3b). This suggests that *R* varies with depth within the lowermost mantle. Despite the covariance between *R*_LL_ and *R*_SR_ and the variability of *R*_LL_ and *R*_SR_ for individual modes, the optimal fit of the entire Stoneley mode data set (see Supplementary Table 1) is obtained when *R*_LL_ and *R*_SR_ are both positive (Fig. 3c), with *R*_LL_=+1.7 to +1.9 and *R*_SR_=+0.4 to +0.9 with *H*=0. Since we combine data for several modes, the corresponding maximum probability value of 0.53 is lower than for individual Stoneley modes (maximum probability of 1.0).

**Figure 4:**
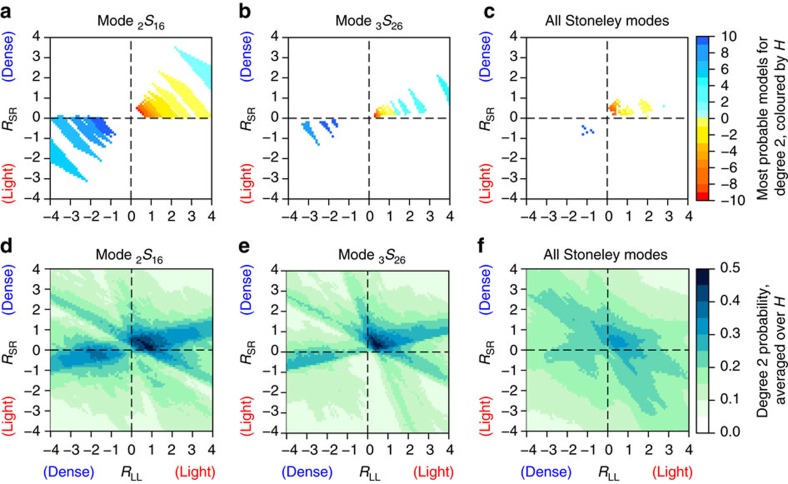
Range of best-fitting density and CMB topography models for Stoneley modes. (**a**,**d**) Stoneley mode _2_*S*_16_. (**b**,**e**) Stoneley mode _3_*S*_26_. (**c**,**f**) All Stoneley modes together. In the top row, only models are shown for which the probability is higher than a threshold of (**d**,**e**) 0.85 or (**f**) 0.50, coloured by the CMB topography scaling factor *H*. Negative values of *H* (yellow/red colours) correspond to dynamically feasible models. The bottom row gives the average probability of the density models when averaged over all possible values of *H*.

From hereon, we focus on the results of the three-parameter model space search, where CMB topography variations are included through the scaling factor *H*. For negative values of *H*, we observe similar patterns in the probability plots of Stoneley modes as before; larger values of *H* give rise to best-fitting models with smaller, but still positive values of *R*_LL_ and *R*_SR_ (top three rows of Fig. 3d–f). These trends are indicative of the trade-off between CMB topography and lowermost mantle density structure^28^. High probability values are confined to a relatively narrow range of *R*_LL_ and *R*_SR_ for large values of *H*, and this widens for lower values of *H*. For positive values of *H*, the pattern shifts quadrants and high probabilities are observed for negative values of both *R*_LL_ and *R*_SR_ (bottom two rows of Fig. 3d–f). Hence, upon including CMB topography, two classes of successful density models emerge. In one class of models, *R*_LL_ and *R*_SR_ are positive and *H*<0, indicating a correlation between density and topography variations. Hence light LLSVPs cover an elevated CMB. In the second class of models, *R*_LL_ and *R*_SR_ are negative and *H*>0, corresponding to an anti-correlation between density and topography variations. In this case, the CMB is elevated below dense LLSVPs instead. The same two classes of models are observed in Fig. 4, where we collapse the three-dimensional parameter space of *R*_LL_, *R*_SR_ and *H* into an two-dimensional space using either a threshold probability or an average probability. Models with *H*<0 have light LLSVPs (yellow/red colours in Fig. 4a–c), whereas models with *H*>0 feature dense LLSVPs (blue colours in Fig. 4a–c). The same two classes of models are present in the average probability plots for individual Stoneley modes (Fig. 4d,e), but for all Stoneley modes combined the highest average probability is found for density models with light LLSVPs (Fig. 4f).

Two opposite classes of density models are compatible with our seismological observations, but we rule out one model class on the basis of geodynamical considerations. For a deformable boundary such as the CMB, long-wavelength structures (∼1,000 km) are expected to be primarily isostatically compensated^30^, implying that *H*<0. Even though density structures throughout the mantle contribute to the CMB topography, tomography-based models of CMB topography strongly correlate with the seismic velocity structure of the lowermost mantle^31^. Thus, we assume that the CMB topography is primarily due to isostatic compensation of long-wavelength density anomalies in the lowermost mantle, so that *H*<0 is expected. Hence, we reject the second class of models with *R*_LL_<0 and *H*>0. This is further justified by the fact that both the model with the highest absolute probability as well as the model with the highest average probability are characterized by *H*<0 and light LLSVPs.

The most probable model with a maximum probability of 0.57 found in the three-parameter model space search (Fig. 4c) is characterized by *R*_LL_=+0.9, *R*_SR_=+0.2 and a topography scaling of *H*=−2 (see Table 1). This is equivalent to degree-2 CMB undulations of ±1 km. We ignore the sensitivity of Stoneley modes to outer core structure, as it is unlikely that significant large-scale heterogeneity can be sustained in the rapidly convecting outer core^32^ and the Stoneley mode splitting functions show a dominantly lower mantle signal^24^. Modelling the higher structural degrees using the same model space search approach produces a possible lowermost mantle density model up to *s*=8 that is compatible with Stoneley mode splitting function measurements (Supplementary Fig. 6). This density model illustrates that not all spherical harmonic degrees are required to have positive values of *R*_LL_ in order for the overall model to still feature light LLSVPs. Table 1 lists the ranges of best-fitting scaling factors, which have a probability within at least 95% of the maximum probability as well as the best-fitting model and its maximum probability. Equivalent values for other threshold levels are reported in Supplementary Table 2 and Supplementary Note 4.

Contrary to previous work^14^,^15^,^16^, our analysis indicates that Stoneley modes are best explained by LLSVPs with a relatively low density. These previous studies are based on the analysis of normal modes that are sensitive to density variations throughout the mantle and have a poorer depth resolution. Moreover, Stoneley mode splitting measurements with a focused sensitivity to the lowermost mantle were missing in these studies, except for mode _1_*S*_14_. We can, in fact, reproduce the previous results (that is, *R*_LL_<0), using either the original whole-mantle mode data set (Supplementary Fig. 7b), or updated measurements for the same modes^23^ (Supplementary Fig. 7c and Supplementary Note 5). In both cases, density models with *R*_LL_<0 and *R*_SR_>0 are most probable (top left quadrant). Using all available splitting function measurements in the current data set^23^,^24^ (see Supplementary Table 1), we cannot determine the sign of *R*_LL_ robustly (Supplementary Fig. 7a). Similar patterns are observed when CMB topography variations are included (see Supplementary Fig. 7d–f). Nonetheless, the best-fitting model in Supplementary Fig. 7d is characterized by *R*_LL_=0.3, *R*_SR_=0.3 and *H*=−10, which is also contained in the range of best-fitting models for the Stoneley mode selection (included in Fig. 4c). By focusing on the Stoneley modes, we are able to extract the signal originating from the deep mantle and we resolve a more pronounced maximum in the probability values.

In previous studies, the focus has been on the longest period normal modes (_0_*S*_2_, _0_*S*_3_, _2_*S*_1_, etc), which also show a large sensitivity to density. The fit of some of these lower mantle sensitive modes (particularly _0_*S*_3_, _0_*S*_7_, _14_*S*_9_) degrades for our best-fitting density models with light LLSVPs (Supplementary Fig. 8), consistent with past normal mode studies^14^,^33^. However, the misfit change of the Stoneley modes is more pronounced, showing that they strongly prefer light LLSVPs. The discrepancy between Stoneley modes and non-Stoneley modes is likely due to unmodelled structure in the mid mantle, which the latter are sensitive to due to their broader sensitivity kernels with depth. When we include these modes, the region of best-fitting models is hence less restricted than when we consider the Stoneley modes only (compare Fig. 3f and Supplementary Fig. 7d). By focusing on the Stoneley modes, whose sensitivity is limited to depths near the CMB, our analysis does not suffer from the known trade-offs with mid and upper mantle structure^20^. Furthermore, this approach allows us to directly explore the sensitivity of the Stoneley modes to lower mantle structure, as opposed to a global-scale inversion of normal mode data for velocity and density structure. Similar approaches are followed in body-wave studies where only core-diffracted and reflected waves are used to study the CMB region, and not upper mantle phases.

The splitting functions of the long-period normal modes are typically constrained by a small number of spectra. Consequently, the uncertainties in the measured coefficients are large, especially in case of mode _0_*S*_2_, for which the splitting function also does not display the ‘Ring around the Pacific' pattern typically observed for lower mantle modes^34^. We find that this mode prefers dense LLSVPs under an L2-norm, consistent with a recent study^33^. However, the uncertainties in its splitting function are so large that any density model is allowed under our probability criterion (Supplementary Fig. 8 and Supplementary Note 6). Hence, _0_*S*_2_ will only raise or lower the total average probability instead of severely affecting the observed region of best-fitting models. On the contrary, the Stoneley mode splitting functions have much smaller uncertainties as they are typically constrained by 2,000–3,000 spectra. Probability estimates based on the Stoneley mode data are hence more robust. Supplementary Fig. 8 provides a strong demonstration of the suitability of the probability criterion over the more commonly used L2-norm.

In summary, the Stoneley modes provide superior resolving power of the density structure in the lowermost mantle and they are not as strongly affected by unmodelled structure in the mid mantle. Thus, we believe that the Stoneley mode splitting measurements are key to resolve the density scaling factor of the LLSVPs.

### Trade-offs with velocity structure

To ascertain that our inferences of light LLSVPs are robust with respect to the assumed velocity structure, we have performed several additional model space searches (see Supplementary Notes 7–10). We emphasize that in these tests we implicitly assume that the long-wavelength shear-wave velocity structure of SP12RTS is representative of the real Earth. However, SP12RTS matches the even-degree structure up to *s*=8 of several recent velocity models very well^35^. Hence, we believe that our results of light LLSVPs are valid for reasonable velocity models and we address further trade-offs between velocity and density towards the end of this section.

We test the assumption of the density scaling factor (*R*=0.3) used in the inversion procedure for SP12RTS in Supplementary Fig. 9. Using a value of *R*=0.0 instead, we obtain very similar density models and systematically find positive values of *R*_LL_ and *R*_SR_, even when no density variations are included anywhere else in the mantle (Supplementary Fig. 9b and Supplementary Note 7). In addition, we have conducted a model space search using SP12RTS_low_D; a lower damped version of SP12RTS (Supplementary Fig. 10), which features larger velocity variations of up to 2% (∼1.4 times larger than in SP12RTS). The predicted splitting function maps (Supplementary Fig. 11) and the results of the model space search (Supplementary Figs 12 and 13) show that density models with positive values of *R*_LL_ still have the highest probability (see Supplementary Note 8). The best-fitting model with a probability of 0.54 for all the Stoneley modes together is characterized by *R*_LL_=1.5, *R*_SR_=−0.9 and *H*=0. Even though this particular model features a negative *R*_SR_, we would like to point out that this test only served to demonstrate that we still find positive values of *R*_LL_ when we increase the amplitude of the shear-wave velocity variations. Furthermore, the best-fitting models for individual Stoneley modes have positive values of *R*_SR_ (Supplementary Figs 12a,e and 13a,b). The maximum probability of the Stoneley modes is lower than when SP12RTS is used, but the probability values of other mode selections are slightly higher (compare Supplementary Fig. 12 with Fig. 3 and Supplementary Fig. 7). The fact that the probability increases when alternative models for the mid mantle are considered, indicates that unmodelled structure at these depths is a likely cause for the discrepancy between Stoneley modes and other lower mantle sensitive modes (Supplementary Fig. 8).

We have investigated the influence of the P-wave velocity structure by relating dln*V*_P_ to dln*V*_S_ through a depth-dependent scaling, instead of using the P-wave velocity structure of SP12RTS (see Supplementary Note 9). This results in a substantially different P-wave velocity model in the lower mantle (SP12RTS_P_scaled) with lower P-wave amplitudes in the lower mantle compared to SP12RTS. Changing the P-wave velocity structure in the mantle has a small effect on the Stoneley modes, as is evident in both the predicted splitting function maps (Supplementary Fig. 14) and the results of the model space search (Supplementary Figs 15 and 16). Generally, we find lower probability values for all mode selections when using SP12RTS_P_scaled (Supplementary Fig. 15), illustrating the strong preference of all normal modes for the P-wave velocity structure of SP12RTS. Nonetheless, the best-fitting model in Supplementary Fig. 16 with a probability of 0.52 is still characterized by positive density scaling factors with *R*_LL_=0.4, *R*_SR_=0.6 and *H*=−7, leading to degree-2 CMB undulations of ±2.8 km. These larger CMB topography variations are needed to compensate for the lower P-wave amplitude as well as the lower values of *R*_LL_. Despite the fact that these topography variations are larger than for SP12RTS, they still obey the criterion of <5 km peak-to-peak topography^28^. Furthermore, when we set *H*=−2 (as found for the best-fitting density model for SP12RTS), the highest probability is found for *R*_LL_=1.0, that is, still lighter LLSVPs. Although we acknowledge that these experiments do not replace a full model space search of velocity and density structure (see Supplementary Note 10), they indicate that the Stoneley modes strongly prefer low overall densities for the LLSVPs.

Finally, when we fully relax the constraints on the velocity structure (for example, remove the dependency on SP12RTS entirely), we observe that the Stoneley modes alone cannot constrain both velocity and density structure. Nonetheless, individual Stoneley modes consistently prefer a positive correlation between density and shear-wave velocity for the two important spherical harmonic coefficients that make up the ‘Ring around the Pacific' structure. This indicates that independent of the assumed shear-wave velocity structure, the Stoneley modes prefer light LLSVPs. To constrain all density and velocity structure coefficients, the Stoneley modes need to be combined with other mantle sensitive modes in a full model space search of whole mantle structure.

We have prescribed constant values of *R*_LL_ and *R*_SR_ for structure below 2,500 km, but these parameters may vary with depth. Experiments in which we introduce a lower layer where *R*_LL_ can vary independently, indicate that for mode _2_*S*_16_ the probability remains the same for both positive and negative values of this lower scaling factor (see Supplementary Note 11 and Supplementary Table 3). For all Stoneley modes combined (Supplementary Table 4), *R*_LL_ must increase to compensate for the lower dense layer, and the resulting probability is slightly lower. This indicates that the Stoneley modes are primarily sensitive to the total heterogeneity in the lowermost mantle, with a trade-off between the depth, thickness and scaling factor in the layer. We observe that it is difficult to constrain the depth variation of the density structure, and in particular the lowest 100 km of the mantle. Nevertheless, the Stoneley modes prefer overall lighter LLSVPs, which is in direct contrast to previous normal mode studies that have found overall dense LLSVPs below 2,500 km depth.

Finally, we regard it unlikely that outer core structure (to which the Stoneley modes are also sensitive) severely alters the inferred best-fitting density models as dynamical considerations limit any lateral density variations to <0.01% (ref. 32). Similarly, patches of ultra-low-velocities found just above the CMB^36^ would need to be significantly larger than typically observed before they would have a significant effect on normal mode data^28^.

## Discussion

The density variations in the most probable model vary from −0.88% within the LLSVPs to +1.10% in the surrounding regions (Supplementary Fig. 6). This resolved density contrast is consistent with previous models that estimate density variations between 0.8 and 1.8% (refs 14, 15, 16), albeit that we find *R*_LL_ for the LLSVPs to be positive for *s*=2. Although we find a relatively high scaling factor of 0.9 for the LLSVPs, the radially averaged dln*ρ*/dln*V*_S_ value is 0.66 as estimated from the median of the dln*ρ*/dln*V*_S_ distribution (Supplementary Fig. 6). Similar high scaling factors have been suggested by previous normal mode studies that found a lower bound of 0.6 (ref. 37) or values between 0.8 and 1.7 (ref. 16). These values are significantly larger than those generally assumed for purely thermal variations (0.2–0.4) (ref. 17).

Current mineral physics estimates allow several possible interpretations. Firstly; in the presence of the lower mantle post-perovskite phase^38^, larger scaling factors of 0.46–0.71 can be obtained for purely thermal variations^39^ (see Methods). Although post-perovskite is expected to occur primarily in the colder regions due to the positive Clapeyron slope of the phase transition, it is still unknown whether it occurs within the LLSVPs as well^3^. The stability field remains under debate, and it is very dependent on the chemical composition^40^ and the CMB temperature^41^. Most observations of anisotropy and seismic discontinuities, commonly attributed to the phase transition, have been made outside the LLSVPs, but some have been reported within the LLSVPS^42^,^43^,^44^,^45^. In addition, studies predict the transition to occur up to 150 km above the CMB within the LLSVPs for reasonable Clapeyron slope values^46^ and patches of post-perovskite material are present inside the LLSVPs in recent geodynamic models^41^. Post-perovskite has also been invoked to explain the observed negative correlation between dln*V*_S_ and dln*V*_C_ inside the LLSVPs^12^,^27^ and observations of core-diffracted waves^29^. If post-perovskite is present both outside and inside the LLSVPs, the predicted scaling factors include our radially averaged value of 0.66. Using upper and lower bounds on the mineral physics derivatives for temperature while post-perovskite is present^39^, the amplitudes of our density variations suggest the LLSVPs to be 550–740 K hotter than the radial average. Similar excess temperatures are obtained in isochemical models of mantle convection^12^.

Alternatively, chemical variations need to be invoked to explain our density scaling factors. While negative scaling factors (dense LLSVPs) always require chemical heterogeneity, our positive scaling factors (corresponding to overall light LLSVPs) cannot be uniquely interpreted (see Methods). Iron enrichment will always lead to higher densities, but variations in mid-ocean-ridge basalts (MORB) and (Fe,Mg)-perovskite can give rise to both lower and higher densities (both positive and negative scaling factors)^39^. Therefore, different combinations of iron-enriched material combined with variations in (Fe,Mg)-perovskite and high temperatures, and possibly post-perovskite could produce the observed range of density variations. Part of the heat to balance high densities due to iron enrichment may originate from heat-producing elements, suggested to be present in the deep mantle from the analysis of Sm/Nd isotopes^47^. Whether the LLSVPs indeed contain large quantities of these elements can be tested by determining regional variations in geoneutrino flux^48^ with forthcoming deployments of geoneutrino detectors in the oceans^49^. This complex balance between thermal and chemical contributions to density anomalies implies that we cannot rule out chemical heterogeneity within the LLSVPs.

The best-fitting *R*_SR_ values of 0.2–0.4 (see Table 1) match the range predicted for purely thermal variations without post-perovskite^17^. However, it is more likely for post-perovskite to be present in these regions, in which case our *R*_SR_ values are lower than the expected range of 0.46–0.71 (see Methods). To still explain our low *R*_SR_ values, we suggest the presence of MORB in these areas, which is a plausible scenario if the faster, surrounding regions represent subducted material.

It may appear that our inferred low-density LLSVPs are inconsistent with their possible long-term stability suggested on the basis of moment-of-inertia considerations^50^ and the correlation between large igneous provinces, kimberlite locations and the reconstructed margins of the LLSVPs^51^. However, LLSVPS morphologies resembling those observed seismically are readily produced by isochemical models of mantle convection, where the flow is entirely driven by plate reconstruction models for 300 Myr (ref. 12), indicating that the present-day LLSVPs can be a result of the overall subduction history and mantle flow instead of anchoring the prevailing flow pattern. Additionally, recent studies have challenged the long-term stability of the LLSVPs^52^,^53^, indicating the need for stronger seismological constraints.

Our analysis indicates that for reasonable velocity models the LLSVPs have an overall low density, but we cannot constrain the sign of *R*_LL_ and *R*_SR_ in the lowest 100 km of the mantle. It is therefore possible that the LLSVPs have a denser-than-average root, possibly due to iron enrichment in the lowermost part of the mantle. The possibility of a denser, likely compositionally distinct root offers an alternative mechanism for the LLSVPs to retain their suggested long-term stability, similar to the scenario recently suggested for broad plumes in the lower mantle^54^. Thermochemical LLSVPs would generally be passively deformed by subducting slabs in the deep mantle while free to migrate along the CMB^8^,^55^. The presence of a denser, compositionally distinct root can anchor the LLSVPs to the CMB, making them long-term stable^4^,^56^. Similar overall positively buoyant or neutrally buoyant LLSVPs with a smaller compositional component towards the deeper parts are consistent with recent geodynamic studies^57^,^58^. Whether overall light, but thermochemical LLSVPs rise, cool down and subsequently sink due to their intrinsic higher density, as observed in simulations of periodically rising and collapsing thermochemical superplumes^7^, will depend on the precise contributions of the thermal and chemical components to the overall density.

Irrespectively whether the observed density scaling factors are due to post-perovskite or chemical heterogeneity, the overall low density of the LLSVPs suggests a component of active present-day upward motion. Their low density also explains the excess-ellipticity of the core^25^ and uplift of the surface^26^,^59^. In either case, the LLSVPs are expected to be lighter with respect to the radial average at those depths if the surrounding regions consist of colder (hence denser) subducted material^3^. The implications of overall low-density LLSVPs therefore need to be investigated with scenarios in which two antipodal regions of the mantle are buoyant. In particular, the dynamic consequences of pPv occurring both inside and outside the LLSVPs and the possibility of a dense basal structure to overall light LLSVPs should be considered. While the Stoneley modes show strong evidence for overall light LLSVPs, future studies should aim to resolve the discrepancy with other lower mantle sensitive modes. For this purpose, as well as for verification of the current results, full model space searches of whole mantle velocity and density structure should be performed.

## Methods

### Normal mode splitting

Earth's normal modes are standing waves arising along the surface and radius of the Earth after large magnitude earthquakes. These oscillations exist only at discrete frequencies due to the finite size of the Earth. Spheroidal mode multiplets _n_*S*_l_ involve P-SV motion and are characterized by their radial order *n* and angular order *l*. For a simple Earth model such as the Preliminary Reference Earth Model or PREM^60^, each multiplet _n_*S*_l_ consists of 2*l*+1 degenerate singlets with azimuthal order *m* in the range −*l*, ..., *l*. Splitting of these singlets into different frequencies occurs due to Earth's rotation, ellipticity and heterogeneous structure.

The splitting of normal modes can be completely described using the generalized splitting function approach^19^. Splitting functions are linearly related to the heterogeneous and anisotropic structure of the Earth dln**m**_st_(*r*), where dln**m** describe the perturbations in velocity, density and anisotropy at radius *r* and spherical harmonics of angular order *s* and azimuthal order *t*, as well as to internal topography variations dln*h*_st_ on discontinuities *d*. Splitting function coefficients *c*_st_ are then given by


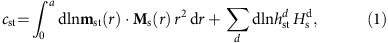


where **M**_s_(*r*) and 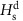 are the associated sensitivity kernels^61^, calculated for PREM. These coefficients, in combination with complex spherical harmonics^62^, are used to visualize how a particular normal mode sees the depth-averaged structure of the Earth.

### Normal mode data

We focus our analysis on splitting function measurements of spheroidal modes, which are sensitive to variations in density and topography in addition to shear- and compressional-wave velocity. We make use of the most extensive splitting function data set available to date (DRH13+KDR13) (refs 23, 24), with frequencies up to 10 mHz (Supplementary Table 1). Both these splitting function data sets are derived from normal mode spectra for 93 large earthquakes with *M*_w_≥7.4 between 1976 and 2011 (ref. 23).

The DRH13 data set consists of 164 modes, including 33 new modes sensitive to compressional-wave velocity variations^23^ and the KDR13 data set contains nine Stoneley modes. From this data set, we exclude modes with sensitivity to the inner core as they are strongly split due to inner core anisotropy^63^. The combined KDR13+DRH13 data set includes additional fundamental modes^64^, producing a data set of splitting measurements for a total of 146 mantle sensitive modes. Comparison of their observed and predicted splitting functions allows us to constrain the range of possible density scaling factors for the LLSVPs. We also show results for the HT96+RR98 data set, which includes 62 modes that were measured before 2000 (refs 18, 19). This older data set formed the basis of previous normal mode density studies^14^,^15^.

We focus our analysis on recent splitting measurements of CMB Stoneley modes^24^, from hereon simply called Stoneley modes and denoted by KDR13 (Stoneley). These modes are uniquely sensitive to structures in the lower mantle and outer core (Supplementary Fig. 3). Stoneley modes are characterized by low overtone numbers and high angular orders. Their sensitivity to the CMB increases with increasing frequency (that is, angular order) (Supplementary Fig. 3a–e) and they have significant sensitivity to density variations near the CMB^24^. Comparison with the sensitivity kernels of another lower mantle sensitive mode (Supplementary Fig. 3f) illustrates the superior sensitivity and depth resolution of Stoneley modes to lowermost mantle structure.

### Density input models

Mantle model SP12RTS (Supplementary Fig. 1) is a long-wavelength model of shear-wave dln*V*_S_ (=*δV*_S_/*V*_S_) and compressional-wave dln*V*_P_ velocity variations in the mantle, derived from P and S body-wave travel times, surface-wave dispersion and normal mode splitting function measurements. The long-wavelength structure of SP12RTS is practically identical to that of shear-wave velocity model S40RTS (ref. 1). SP12RTS contains many features observed in other tomographic models, including large-low shear- and compressional-wave velocities underneath the Pacific and Africa in the lower mantle (Supplementary Fig. 1a,b), a high ratio of dln*V*_S_ to dln*V*_P_ variations and an anti-correlation between shear-wave velocity and bulk-sound velocity variations. The LLSVPs (or ‘LL') are surrounded by a ring of higher velocities, possibly related to the accumulation of subducted material (denoted here as ‘surrounding regions' or ‘SR'). Predicted splitting functions are obtained for SP12RTS assuming that density variations dln*ρ* are the same as dln*V*_S_ except for a scaling factor *R*=dln*ρ*/dln*V*_S_. If, for example *R*<0, the density is relatively high in regions with low shear-wave velocities and it is relatively low in high-velocity regions. Model SP12RTS assumes a default value of *R*=+0.3 (ref. 17).

Below 2,500 km depth, we vary the scaling factors *R*_LL_ and *R*_SR_ between values of −4 and 4 (±5% density variations), which describe the density variations in the LLSVPs (defined by dln*V*_S_<−0.10%) and the surrounding regions (defined by dln*V*_S_>0.50%), respectively (Fig. 1b). These two regions coincide approximately with areas of different *R* values found in previous density models^65^. Using the −0.10% contour, the ‘LL' regions coincide with the extent of the LLSVPs as found in a lower mantle clustering analysis (Fig. 1a)^2^. Although the −0.1% shear-wave velocity contour is lower than generally used as definition for the LLSVPs, it matches the commonly used −1.0% contour of the SMEAN model^66^, as the velocity amplitudes of SP12RTS are lower. The contour value of 0.50% for the ‘SR' regions is chosen such that their areal extent is similar to the LLSVPs; both regions now cover about 25% of the CMB, whereas the ‘SR' regions would be significantly larger than the ‘LL' regions if we use the same value (for example, a contour of +0.10%). Outside the ‘SR' and ‘LL' regions, the default scaling factor of 0.3 is used. However, as the shear-wave velocity anomalies in these areas are low, this only results in small density variations, which do not contribute much to the overall density structure (variations <0.15%). Experiments in which we perform a model space search of all five coefficients for structural degree *s*=2 produce extremely similar best-fitting density models, indicating that our parameterization in *R*_LL_ and *R*_SR_ is justified. Supplementary Fig. 2 shows some extreme examples of density input models for different values of *R*_LL_ and *R*_SR_. In addition, we vary the depth below which these two scaling factors are changed between 2,300 and 2,800 km (Supplementary Tables 3 and 4). Furthermore, we investigate the possibility of the LLSVPs being raised above the CMB, with an additional scaling factor 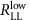 used to describe the density variations of the LLSVPs in the lower layer.

CMB topography variations dln*h*_CMB_ are included by scaling them to lower mantle density variations using the scaling factor *H*=dln*h*_CMB_/dln*ρ*. CMB topography variations are the consequence of density variations that are isostatically compensated in combination with dynamic stresses due to mantle flow. For a deformable boundary such as the CMB, we expect long-wavelength structures (∼1,000 km) to be almost entirely isostatically compensated^30^, leading to negative values of *H*, meaning a depressed CMB under dense LLSVPs and an uplifted CMB under regions of light material. A simple isostatic balance using PREM densities and a layer thickness of 400 km gives *H*≈−5. Nonetheless, we vary *H* between −10 and 10 in order to search the full model space. For negative values of *H*, topography and density are correlated (that is, an elevated CMB where the density is lower) whereas for positive values of *H*, topography and density are anti-correlated (that is, dense LLSVPs cover an elevated CMB).

### Measure of fit

We use a robust statistical approach to assess the performance of the density models using the uncertainties of the splitting function measurements. The probability indicates whether a particular density model fits the observed splitting function coefficients within their uncertainties. The probability *P*_s_ is defined as a normalized conditional sum of inverse uncertainties for those splitting function coefficients that are fit within their uncertainties:


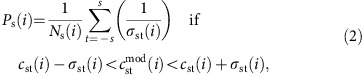


where *c*_st_(*i*) are the observed splitting function coefficients of angular order *s* and azimuthal order *t* for the *i*^th^ normal mode, 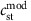
(*i*) the predicted splitting function coefficients for a given density model and *σ*_st_ the uncertainties of the observed splitting function coefficients. The uncertainties *σ*_st_ have been estimated using the maximum spread in observed coefficients in cross-validation runs^23^. *N*_s_(*i*) is a normalization factor, corresponding to the sum of all inverse uncertainties for the *i*^th^ mode at angular order *s*:


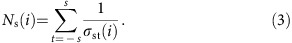


A probability value of 1 implies that all data are fit within their uncertainties and a value of 0 indicates that no coefficients fit. This measure of fit ensures that more emphasis is given to normal modes with well-constrained splitting function coefficients.

In addition to calculating probability values for individual normal modes, we obtain values for selections of normal modes (Supplementary Table 1) by summing over individual mode values, normalized by the number of modes *N* in each selection:


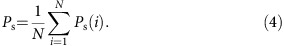


### Mineral physics interpretation

We use recent mineral physics estimates to determine the possible range of density scaling factors expected for thermal and chemical variations in the presence of the lower mantle post-perovskite phase^39^. dln*ρ*/dln*V*_S_ values are obtained by dividing the sensitivities of density dln*ρ*/d*X* and shear-wave velocity dln*V*_S_/d*X*, where *X* denotes variations in temperature, iron, (Mg,Fe)-perovskite and MORB. The given temperature sensitivities are used to estimate the temperature of the LLSVPs, and in each case, the given ranges are bound by the 0.15 and 0.85 quartiles (that is, encompassing 70% of the explored sensitivities). The resulting dln*ρ*/dln*V*_S_ ranges at depths of 2,500–2,900 km are 0.46 to 0.71 for variations in temperature, −1.1 to −0.9 for variations in iron, −2.2 to +1.8 for variations in (Mg,Fe)-perovskite and −2.7 to +1.5 for variations in MORB. Negatively scaling factors hence point uniquely to chemical variations, but positive scaling factors can be obtained in a number of ways.

### Data availability

The splitting function data sets are available as supporting online material with the relevant publications^23^,^24^. The computer codes for calculating splitting functions are available from the corresponding author upon reasonable request.

## Additional information

**How to cite this article:** Koelemeijer, P. *et al*. Density structure of Earth's lowermost mantle from Stoneley mode splitting observations. *Nat. Commun.* **8,** 15241 doi: 10.1038/ncomms15241 (2017).

**Publisher's note**: Springer Nature remains neutral with regard to jurisdictional claims in published maps and institutional affiliations.

## Supplementary Material

Supplementary Information Supplementary Figures, Supplementary Tables, Supplementary Notes and Supplementary References

## Figures and Tables

**Table 1:** Overview of best-fitting scaling factors per structural degree.

	**Range**			**Best fit**			
**Structural degree** ***s***	***R***_**LL**_	***R***_**SR**_	***H***	***R***_**LL**_	***R***_**SR**_	***H***	**Maximum probability**
2	0.9 to 1.3	0.2 to 0.4	−2 to −1	0.9	0.2	−2	0.57
4	−1.7 to 2.3	0.5 to 2.4	−9 to 0	1.9	1.8	0	0.42
6	−3.3 to −1.2	0.6 to 1.3	−10 to −3	−2.2	1.1	−4	0.48
8	3.8 to 3.8	2.0 to 2.0	−3 to −3	3.8	2.0	−3	0.42

Analysis based on Stoneley mode data at each structural degree.

The indicated range reflects the models for which the probability is at least 95% of the maximum probability.
