# Supplementary material for: Density structure of Earth's lowermost mantle from Stoneley mode splitting observations
Source: Nat Commun. 2017 May 15;8:15241. doi: 10.1038/ncomms15241 (PMC5440685; doi:10.1038/ncomms15241)
Supplement: Supplementary Information — Supplementary Figures, Supplementary Tables, Supplementary Notes and Supplementary References [file ncomms15241-s1.pdf]

Supplementary Figures

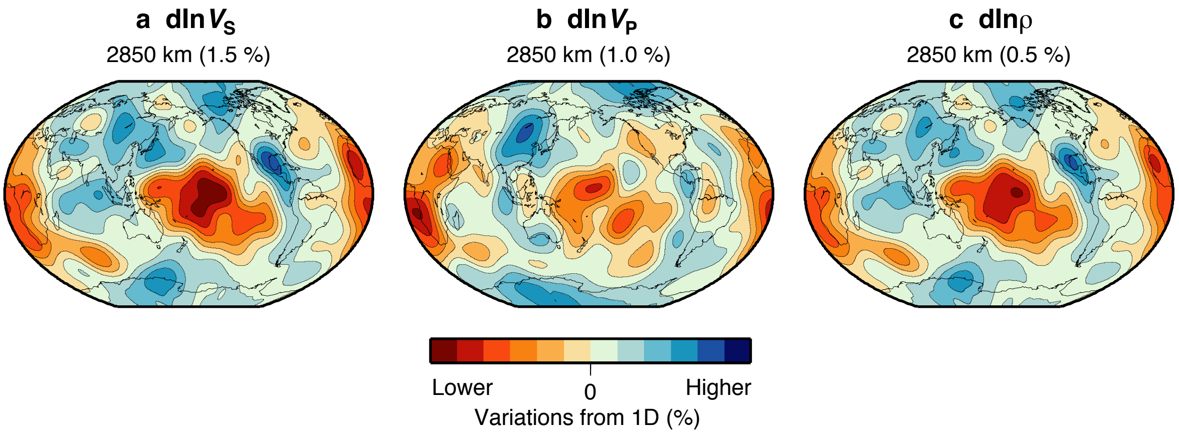

Supplementary Figure 1: Mantle structure at 2850 km depth according to model SP12RTS [1]. **a**, Shear-wave velocity variations dlnV_S. **b**, Compressional-wave velocity variations dlnV_P . **c**, Density variations dlnρ. The density map is a scaled version of the shear-wave velocity map (dlnρ = 0.3 * dlnV_S).

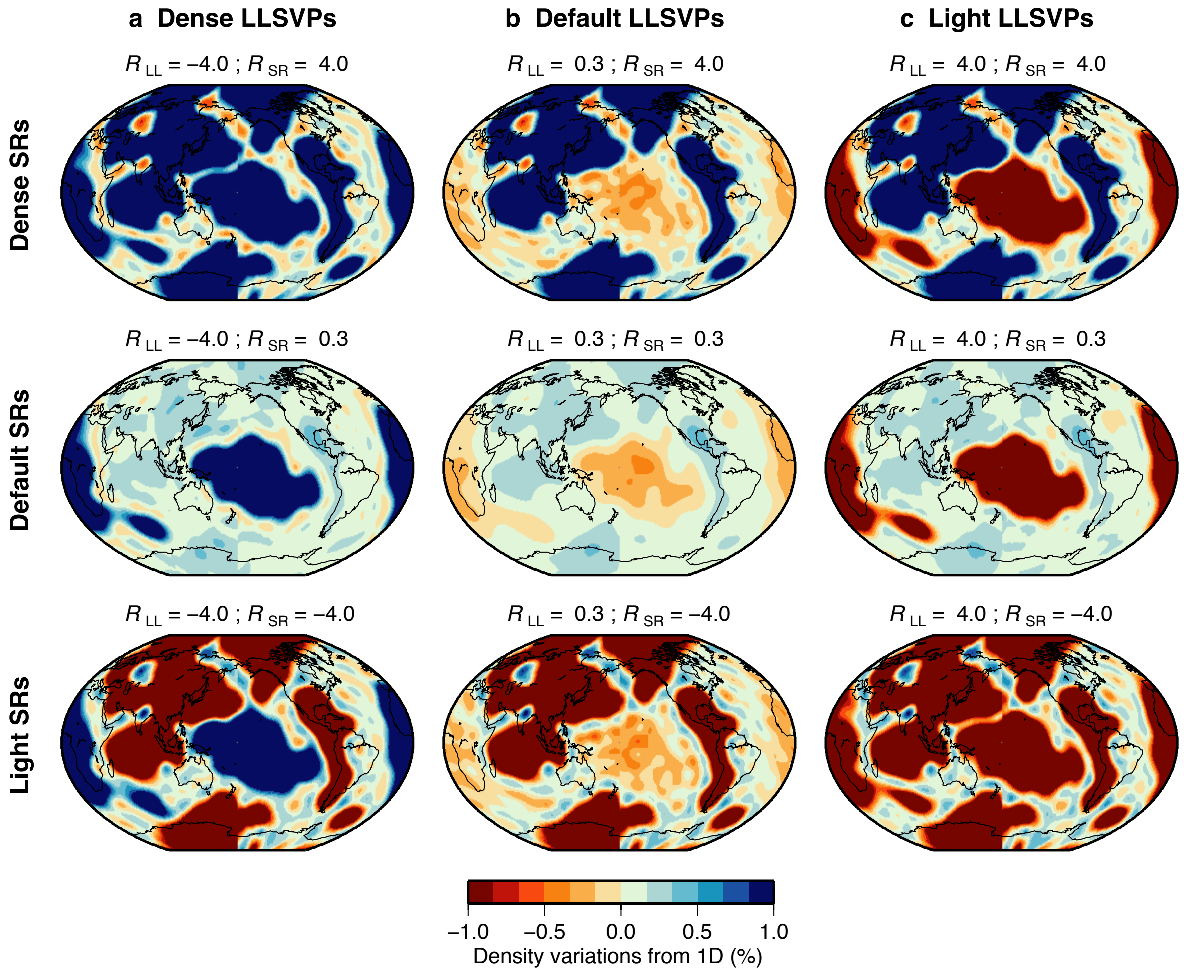

Supplementary Figure 2: Examples of extreme density input models. a, Models with dense LLSVPs (R_{LL} = −4). **b**, Models with default LLSVPs (R_{LL} = 0.3). **c**, Models with light LLSVPs (R_{LL} = 4). The surrounding regions (SRs) contain dense structures for R_{SR} = 4 (top) and light structures for R_{SR} = −4 (bottom). The default model is defined by R_{LL} = R_{SR} = 0.3 (centre).

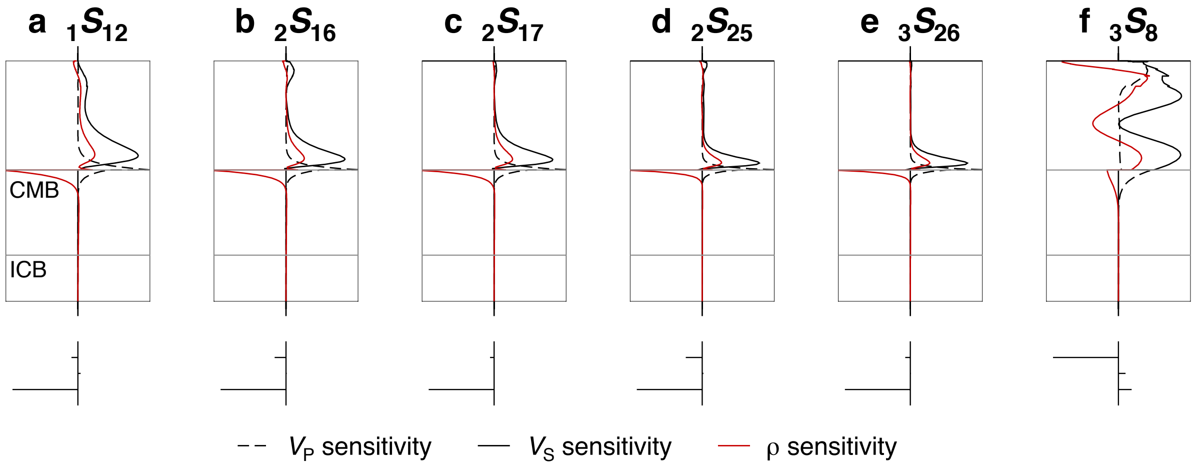

Supplementary Figure 3: Depth kernels describing the sensitivity to density *ρ* (red), shear-wave velocity V_S (solid) and compressional-wave velocity V_P (dashed). a–e, Stoneley modes. **f**, a lower mantle sensitive mode. The radii of the CMB and ICB are indicated by horizontal lines. Horizontal bars underneath the plots show from top to bottom the sensitivity to topography on the free surface, the 660-km discontinuity and the CMB. Each graph is scaled independently. The Stoneley modes have a much more focused sensitivity to structures close to the CMB.

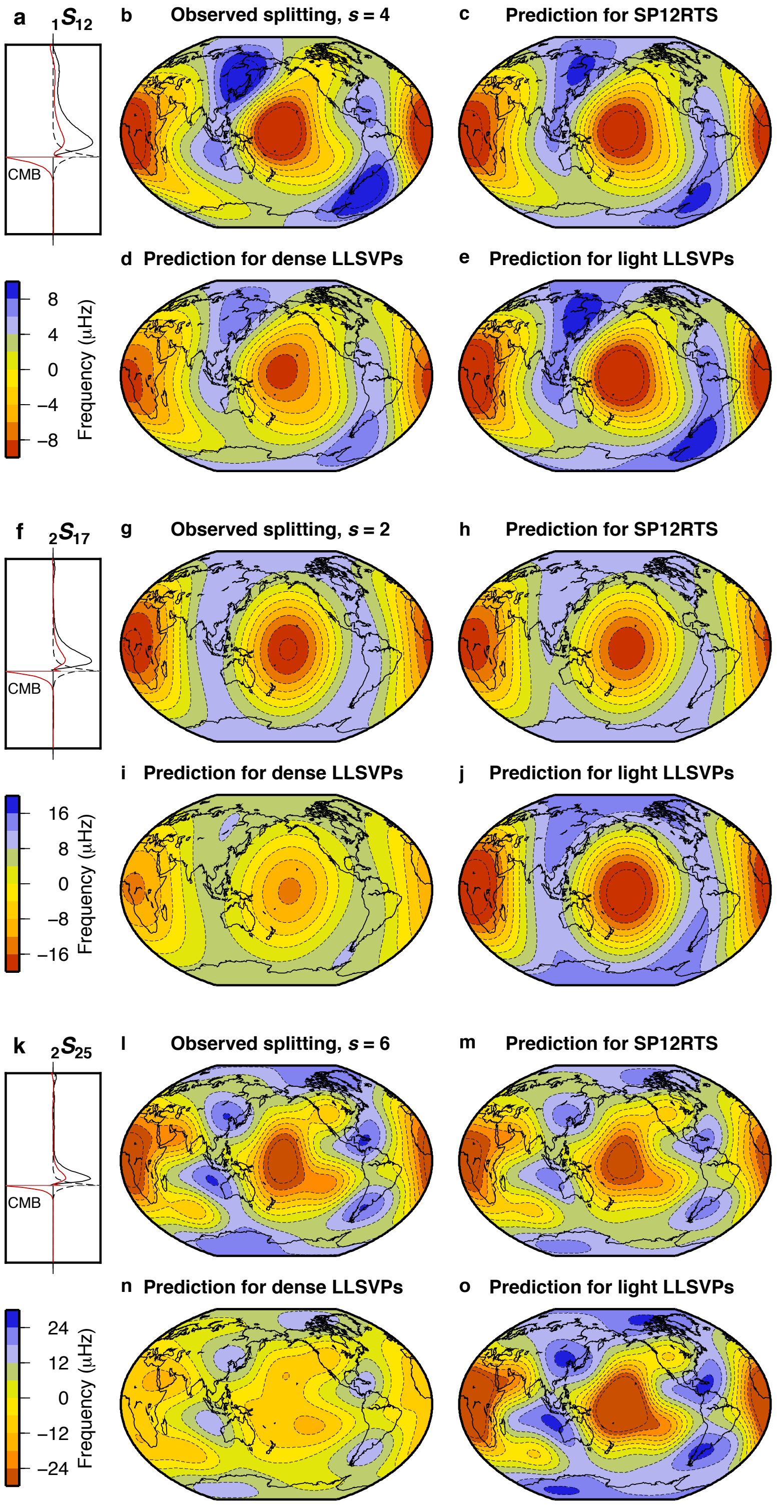

Supplementary Figure 4: Observed and predicted Stoneley mode splitting function maps. a, **f** and **k**, Sensitivity kernels for density (red), shear-wave velocity (solid) and compressional-wave velocity (dashed) structure for modes $_1S_{12}$, $_2S_{17}$ and $_2S_{25}$ respectively. **b**, Observed splitting for $_1S_{12}$ plotted up to maximum structural degree *s*. **c**, Predicted splitting for mantle model SP12RTS [1] and crustal model CRUST5.1 [2]. **d**, Predicted splitting for dense LLSVPs (R_{LL} = −4 and R_{SR} = +0.3). **e**, Predicted splitting for light LLSVPs (R_{LL} = +4 and R_{SR} = +0.3). **g-j**, Similar as **b-e**, but for mode $_2S_{17}$. **l-o**, Similar as **b-e**, but for mode $_2S_{25}$. CMB topography variations are excluded.

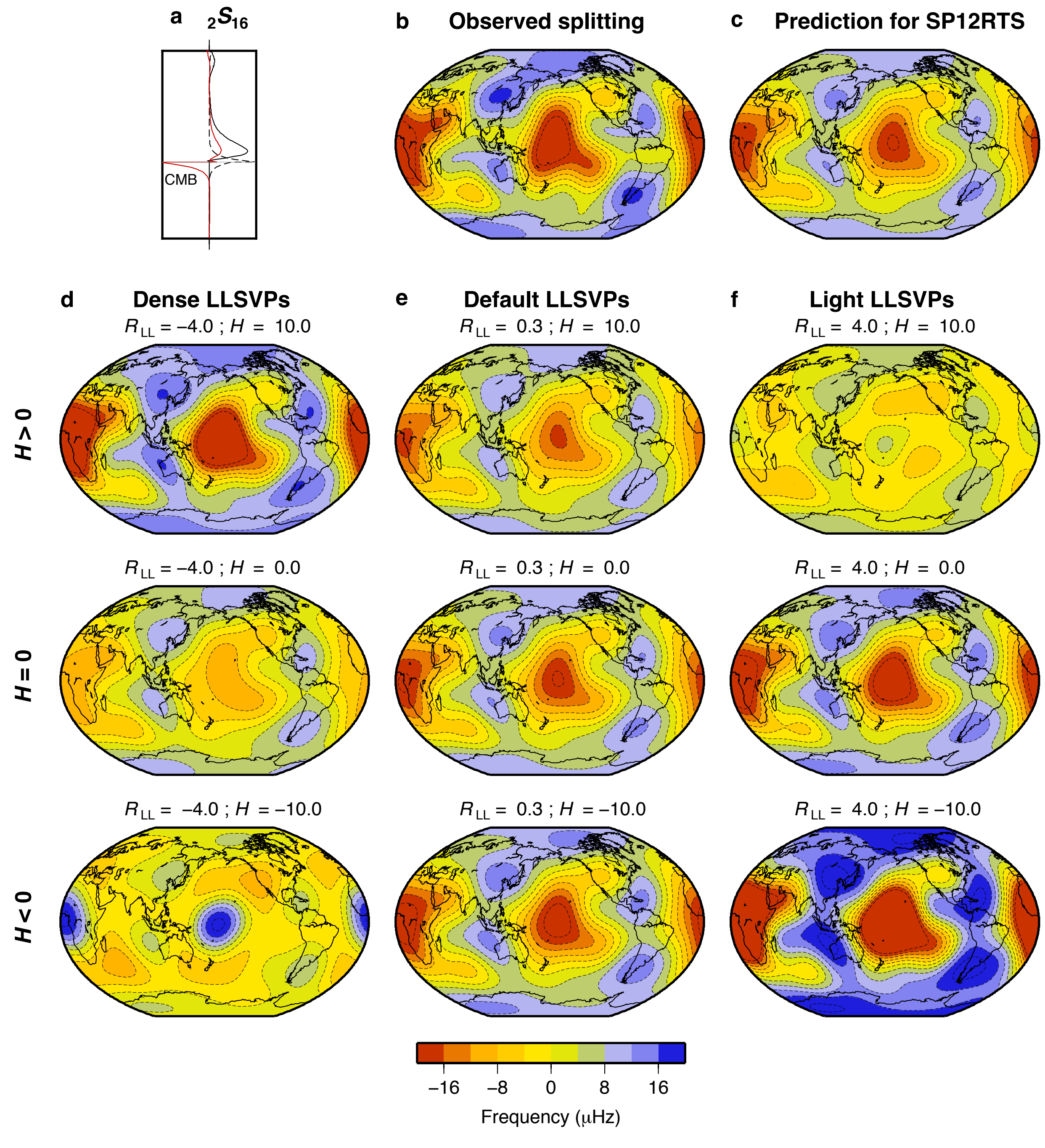

Supplementary Figure 5: Observed and predicted splitting function maps for Stoneley mode $_2S_{16}$. **a**, Sensitivity kernels for density (red), shear-wave velocity (solid) and compressional-wave velocity (dashed) structure. **b**, Observed splitting function plotted up to structural degree s = 6. **c**, Predicted splitting for mantle model SP12RTS [1] and crustal model CRUST5.1 [2]. **d**, Predicted splitting for dense LLSVPs (R_{LL} = −4) with positively (top), no (middle) and negatively (bottom) scaled CMB topography variations (denoted by the scaling factor H). **e**, Similar as **d**, but for default LLSVPs (R_{LL} = +0.3). **f**, Similar as **d**, but for light LLSVPs (R_{LL} =+4).

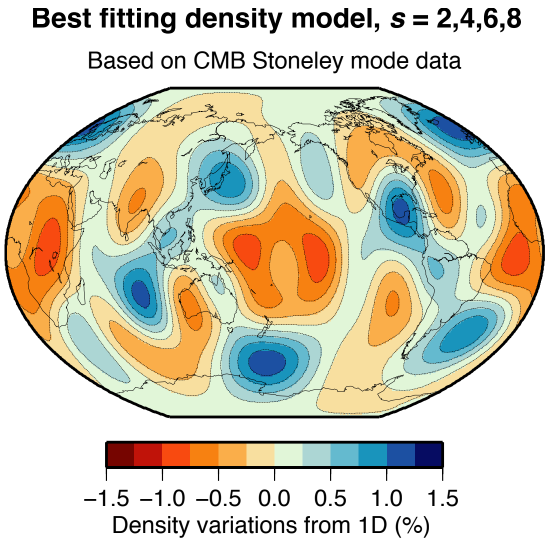

Supplementary Figure 6: Example of a possible lowermost mantle density model up to *s* = 8. This model is determined using the best fitting density scaling factors at each structural degree, based on all Stoneley mode data.

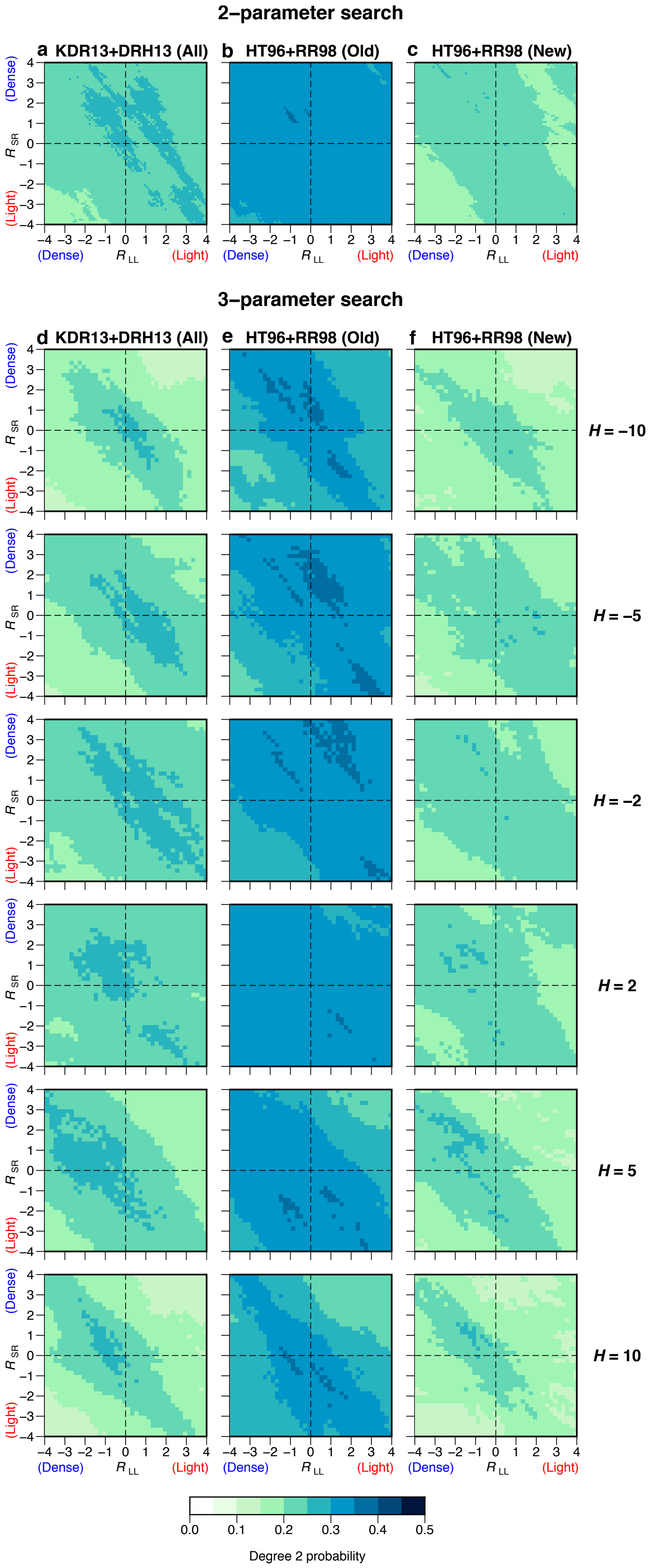

2–parameter search
a KDR13+DRH13 (All)
b HT96+RR98 (Old)
c HT96+RR98 (New)
3–parameter search
d KDR13+DRH13 (All)
e HT96+RR98 (Old)
f HT96+RR98 (New)
H = –10
H = –5
H = –2
H = 2
H = 5
H = 10
R SR
R LL
(Dense)
(Light)
Degree 2 probability

Supplementary Figure 7: Probability of density models for different mode selections. a–c, 2- parameter search without CMB topography variations for **a**, All modes of the KDR13 and DRH13 data sets. **b**, All modes of the old HT96 and RR98 data sets, which were used in previous studies. **c**, All modes of the HT96 and RR98 data sets, using updated measurements from DRH13. **d–f**, Similar as **a–c** but for the 3-parameter search including CMB topography variations. As in Fig. 3 in the main text, the probability of density models is shown for different values of H, the scaling factor between lower mantle density variations and CMB topography, which is indicated on the right of each row. Negative values of H correspond to dynamically feasible models.

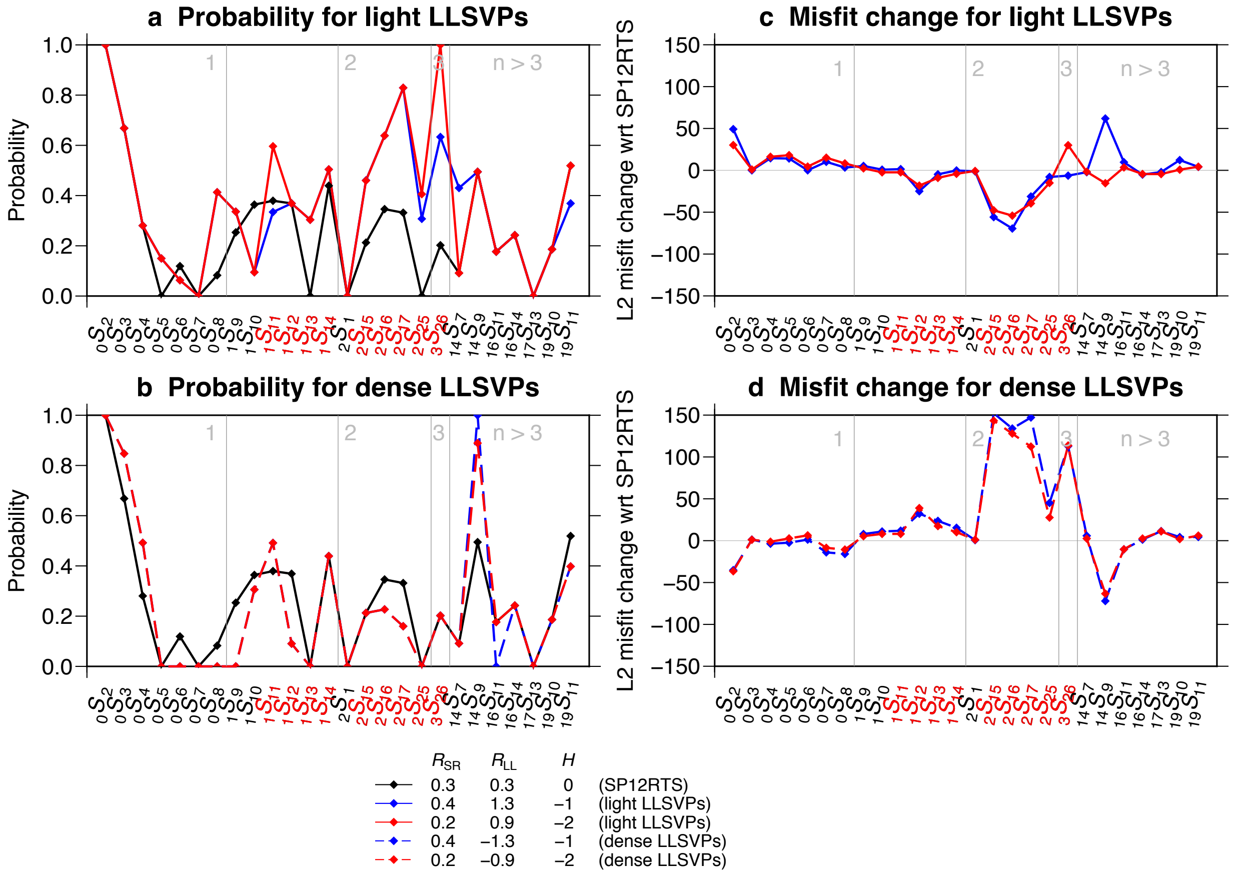

Supplementary Figure 8: Probability and L2-norm changes for lowermost mantle modes. a-b, Probability values and **c-d** L2-norm changes compared to SP12RTS for modes sensitive to shear-wave, compressional-wave and density structure in the lowermost mantle. Probability values and L2-norm changes are shown for the two best fitting models of Table 1, which feature light LLSVPs (solid blue and red lines in **a, c**). We also present the fit of the same models when the sign of R_{LL} is reversed (dashed blue and red lines in **b, d**). Stoneley modes are indicated by red and the probability values of the default density model (with $R_{LL} = R_{SR} = 0.3$) are indicated in black.

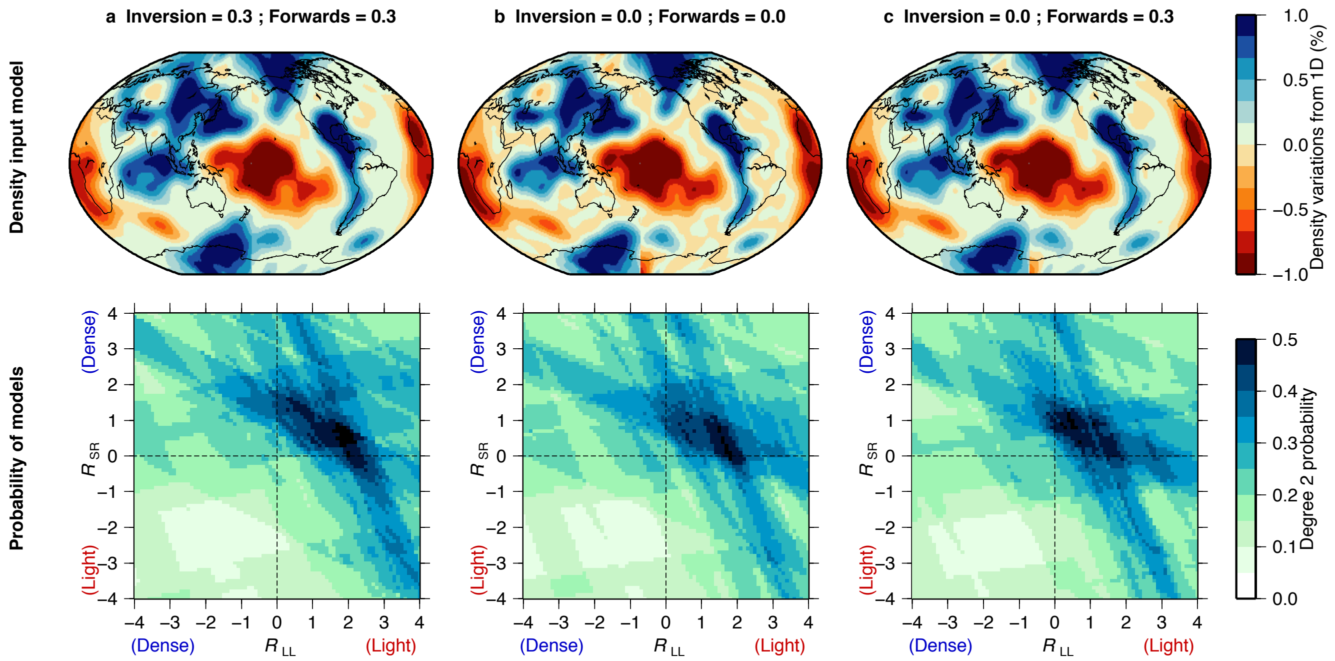

Supplementary Figure 9: Effect of the inversion density scaling factor on our model space search. Examples of density input models with R_{LL} = R_{SR} = 1 (top) and the resulting model space search for the Stoneley mode selection (bottom). **a**, Default density variations (R = 0.3) for the inversion and forward modelling. **b**, No density variations (R = 0.0) in the inversion or forward modelling. **c**, No density variations (R = 0.0) in the inversion, default density variations (R = 0.3) in the forward modelling.

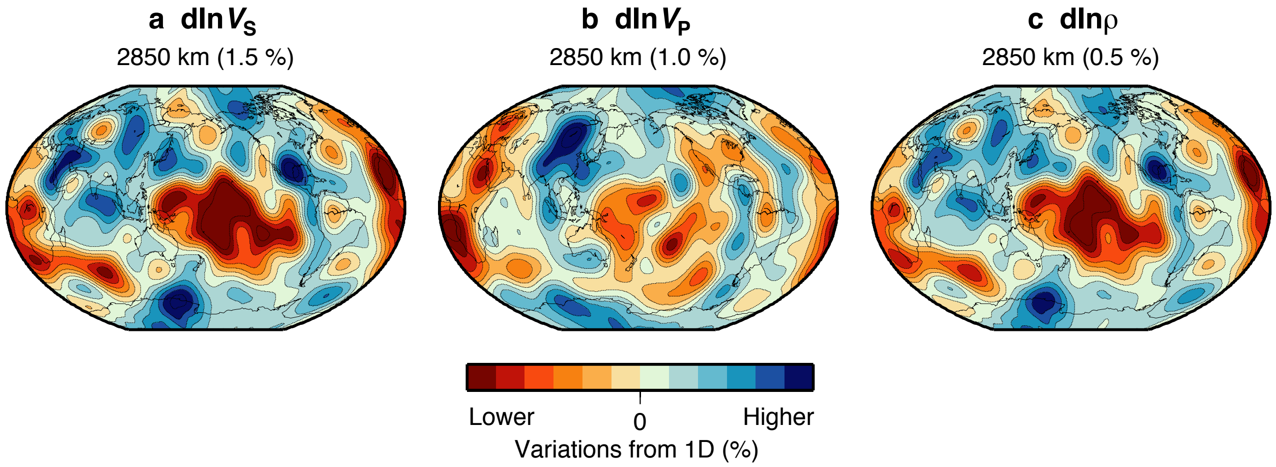

Supplementary Figure 10: Mantle structure at 2850 km depth for model SP12RTS_low_D. a-c, Velocity and density variations as in Supplementary Fig. 1. Note the larger amplitude of the velocity variations in this lower damped version of SP12RTS compared to Supplementary Fig. 1.

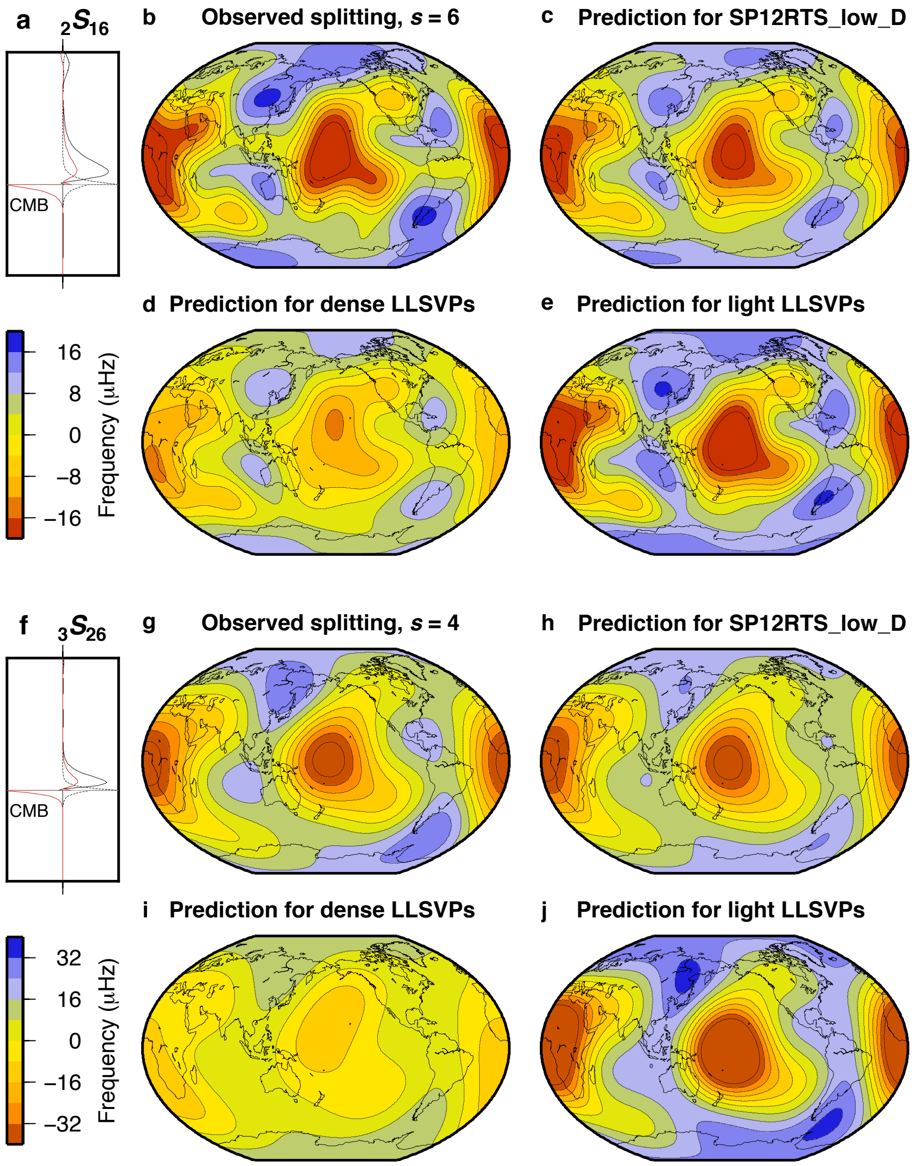

Supplementary Figure 11: Observed and predicted Stoneley mode splitting function maps using model SP12RTS_low_D. a-j, As Fig. 2 in the main text, but velocity variations are now described by a lower damped version of SP12RTS (see Supplementary Fig. 10).

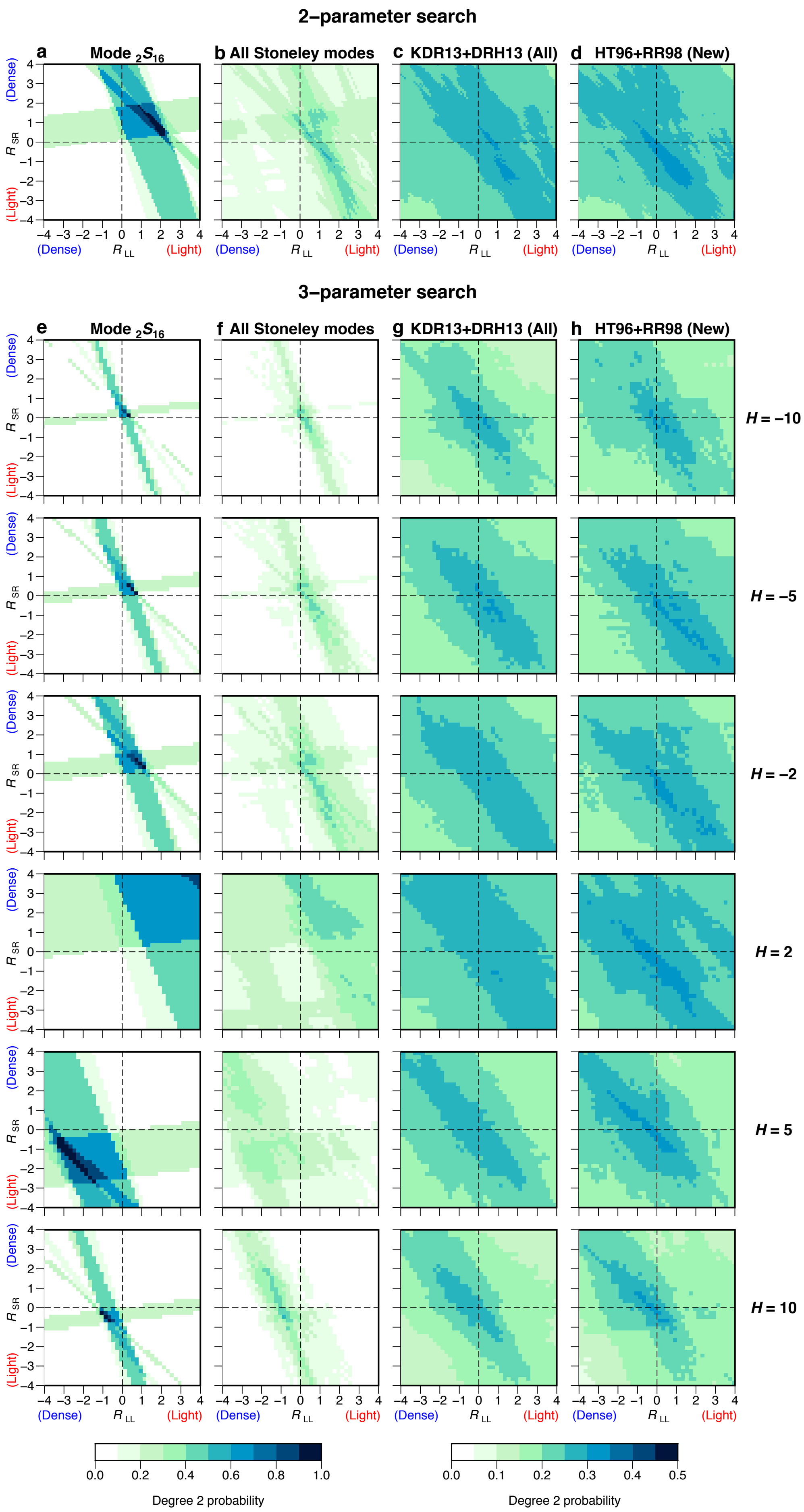

2–parameter search
a Mode $_2S_{16}$
b All Stoneley modes
c KDR13+DRH13 (All)
d HT96+RR98 (New)
3–parameter search
e Mode $_2S_{16}$
f All Stoneley modes
g KDR13+DRH13 (All)
h HT96+RR98 (New)
H = −10
H = −5
H = −2
H = 2
H = 5
H = 10
R_{SR}
(Dense)
(Light)
R_{LL}
Degree 2 probability
Degree 2 probability

Supplementary Figure 12: Probability of density models for individual modes and mode selections using model SP12RTS low D. a–h, As Fig. 3 in the main text and Supplementary Fig 7, but velocity variations are described by a lower damped version of SP12RTS (see Supplementary Fig. 10). Note the difference in scale between the left two and right two columns.

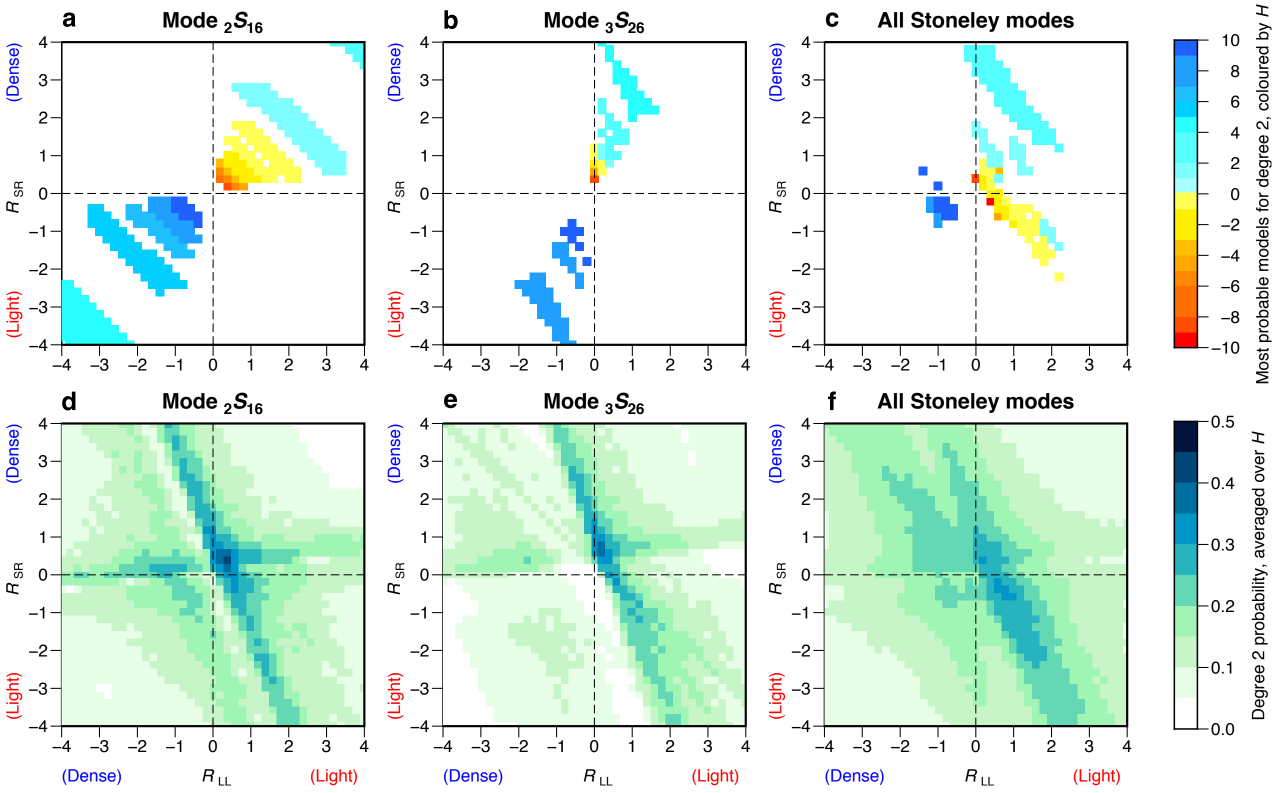

Supplementary Figure 13: Range of best fitting density and CMB topography models for Stoneley modes using model SP12RTS low D. a–f, As Fig. 4 in the main text, but velocity variations are now described by a lower damped version of SP12RTS (see Supplementary Fig. 10). We show models for which the probability is higher than **a–b** 0.80 or **c** 0.45 in the top row, whereas the bottom row shows the average probability of all models averaged over the different values of *H*.

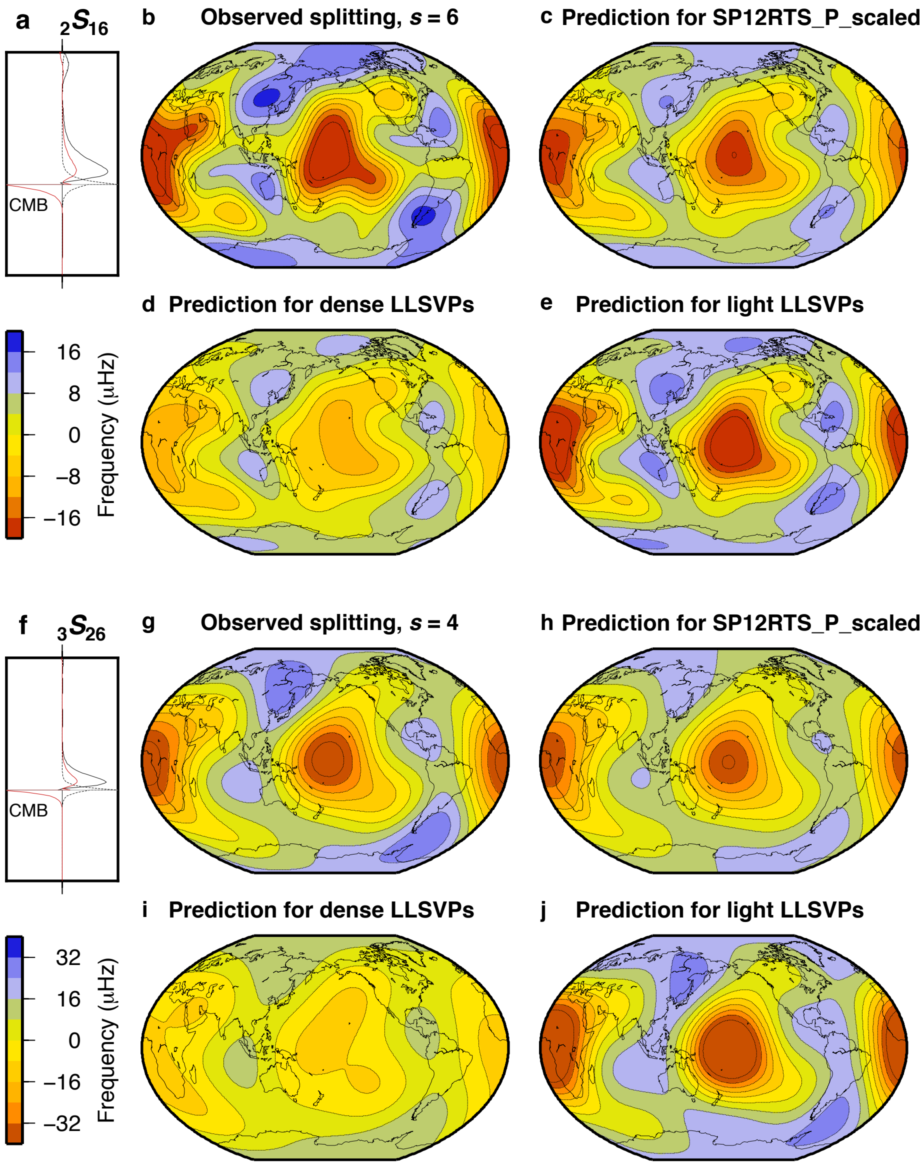

Supplementary Figure 14: Observed and predicted Stoneley mode splitting function maps using model SP12RTS P scaled. a–j, As Fig. 2 in the main text, but dlnV_P are now scaled to the dlnV_S of SP12RTS using a depth-dependent scaling.

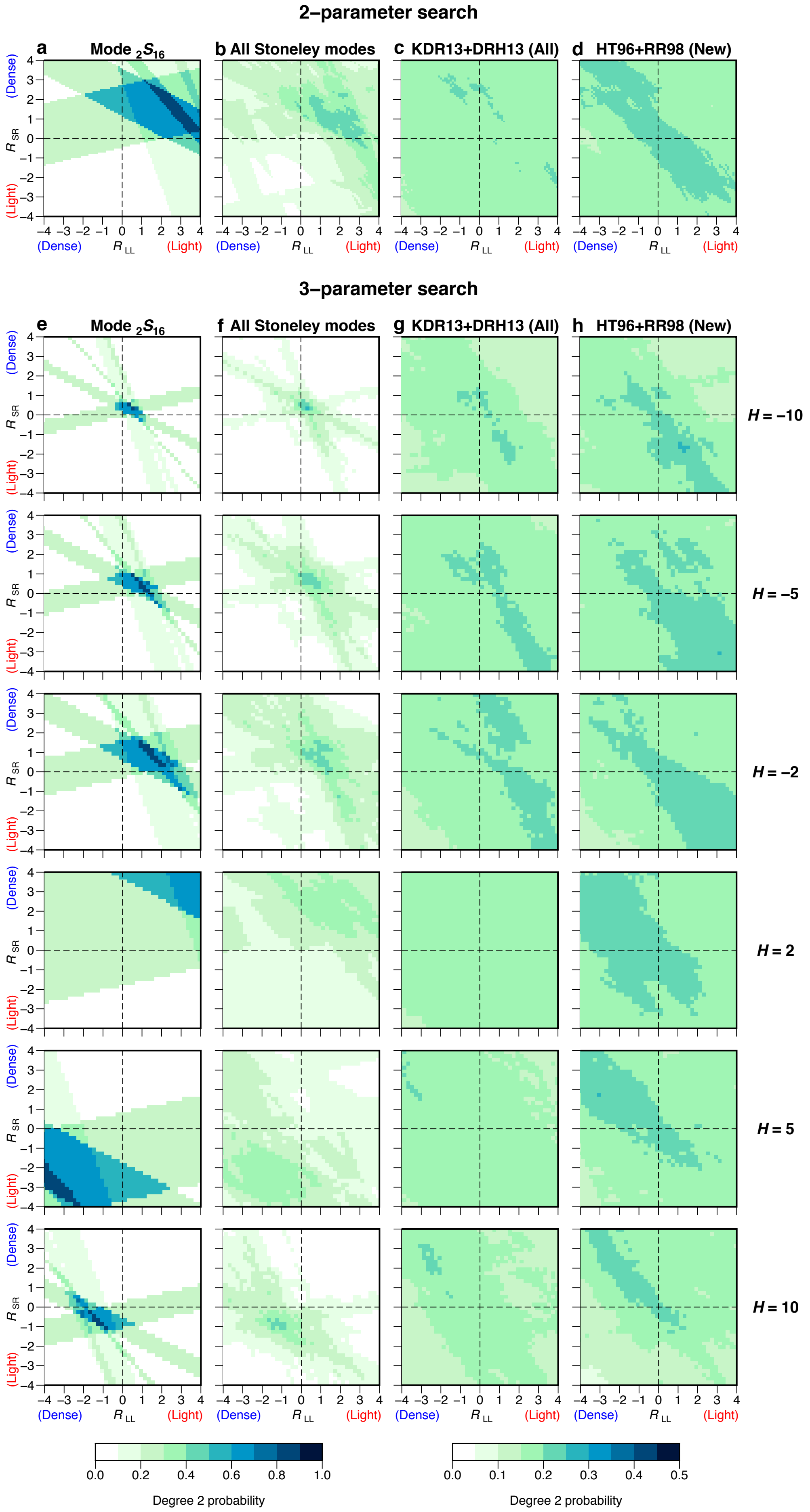
2–parameter search
a Mode $_2S_{16}$
b All Stoneley modes
c KDR13+DRH13 (All)
d HT96+RR98 (New)
3–parameter search
e Mode $_2S_{16}$
f All Stoneley modes
g KDR13+DRH13 (All)
h HT96+RR98 (New)
H = −10
H = −5
H = −2
H = 2
H = 5
H = 10
R_{SR}
(Dense)
(Light)
R_{LL}
Degree 2 probability
Degree 2 probability

Supplementary Figure 15: Probability of density models for individual modes and mode selections using model SP12RTS P scaled. a–h, As Supplementary Fig. 12, but dlnV_P are now scaled to dlnV_S in SP12RTS using a depth-dependent scaling. Note the difference in scale between the left two and right two columns.

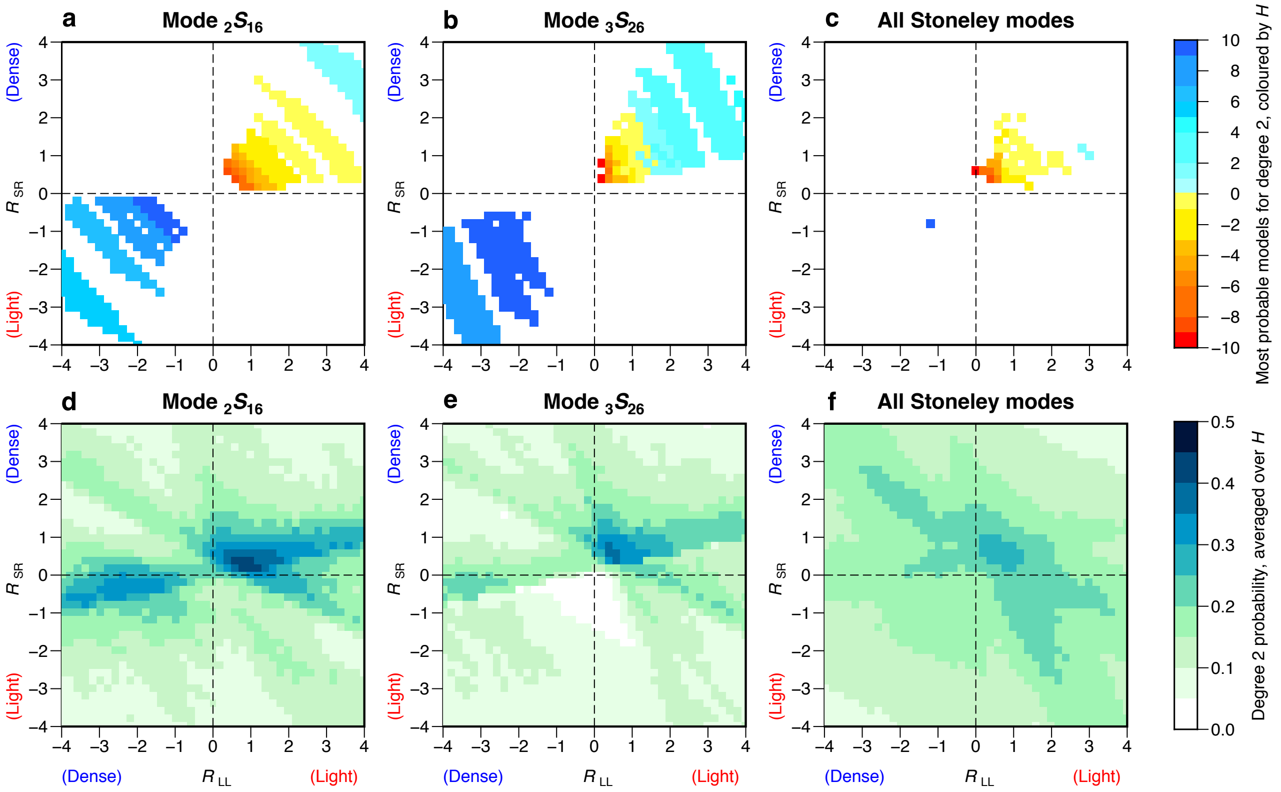

Supplementary Figure 16: Range of best fitting density and CMB topography models for Stoneley modes using model SP12RTS P scaled. a–f, As Fig. 4 in the main text, but dlnV_P are now scaled to dlnV_S in SP12RTS. We show models for which the probability is higher than **a–b** 0.80 or **c** 0.45 in the top row, whereas the bottom row shows the average probability of all models averaged over the different values of H.

Supplementary Tables

Supplementary Table 1: Overview of modes included in the different splitting function data sets.

Selection	*n*	Modes	*n*	Modes
KDR13*(Stoneley)	1	${}_1S_{11}$-${}_1S_{14}$		
	2	${}_2S_{15}$-${}_2S_{17}$, ${}_2S_{25}$		
	3	${}_3S_{26}$		
KDR13+DRH13§	0	${}_0S_2$-${}_0S_9$, ${}_0S_{12}$-${}_0S_{30}$	10	${}_{10}S_{10}$, ${}_{10}S_{17}$-${}_{10}S_{21}$
	1	${}_1S_2$-${}_1S_{16}$	11	${}_{11}S_9$-${}_{11}S_{10}$, ${}_{11}S_{12}$, ${}_{11}S_{14}$, ${}_{11}S_{23}$-${}_{11}S_{25}$
	2	${}_2S_1$, ${}_2S_4$-${}_2S_{17}$, ${}_2S_{25}$	12	${}_{12}S_6$-${}_{12}S_8$, ${}_{12}S_{11}$-${}_{12}S_{17}$
	3	${}_3S_6$-${}_3S_9$, ${}_3S_{25}$-${}_3S_{26}$	13	${}_{13}S_{15}$-${}_{13}S_{16}$, ${}_{13}S_{18}$-${}_{13}S_{20}$
	4	${}_4S_1$-${}_4S_5$	14	${}_{14}S_7$-${}_{14}S_9$, ${}_{14}S_{13}$-${}_{14}S_{14}$
	5	${}_5S_3$-${}_5S_8$, ${}_5S_{11}$-${}_5S_{12}$, ${}_5S_{14}$-${}_5S_{17}$	15	${}_{15}S_{12}$, ${}_{15}S_{15}$-${}_{15}S_{16}$
	6	${}_6S_9$-${}_6S_{10}$, ${}_6S_{15}$, ${}_6S_{18}$	16	${}_{16}S_{10}$-${}_{16}S_{11}$, ${}_{16}S_{14}$
	7	${}_7S_5$-${}_7S_9$	17	${}_{17}S_{12}$-${}_{17}S_{15}$
	8	${}_8S_6$-${}_8S_7$, ${}_8S_{10}$	19	${}_{19}S_{10}$-${}_{19}S_{11}$
	9	${}_9S_6$, ${}_9S_8$, ${}_9S_{10}$-${}_9S_{15}$		
HT96°+RR98¶	0	${}_0S_3$-${}_0S_9$, ${}_0S_{12}$-${}_0S_{21}$	6	${}_6S_{10}$
	1	${}_1S_2$-${}_1S_{10}$, ${}_1S_{14}$	7	${}_7S_6$-${}_7S_7$
	2	${}_2S_4$-${}_2S_{13}$	8	${}_8S_7$
	3	${}_3S_6$-${}_3S_9$	9	${}_9S_{14}$
	4	${}_4S_1$-${}_4S_5$	12	${}_{12}S_8$, ${}_{12}S_{12}$
	5	${}_5S_3$-${}_5S_6$	17	${}_{17}S_{12}$-${}_{17}S_{13}$

* KDR13: Koelemeijer et al., GRL, 2013 [3]. This data set contains nine Stoneley modes.
§ DRH13: Deuss et al., GJI, 2013 [4].
° HT96: He & Tromp, JGR, 1996 [5].
¶ RR98: Resovsky & Ritzwoller, JGR, 1998 [6].
There are 146 modes in the combined KDR13+DRH13 data set and 62 modes in the HT96+RR98 data set.

Supplementary Table 2: Overview of ranges of best fitting scaling factors per structural degree.

Structural degree *s*	R_{LL}	R_{SR}	H	
2	0.4 to 2.2	0.2 to 0.9	-5 to -0	Threshold of 90 %
4	-2.6 to 2.6	-1.7 to 3.6	-10 to 0	
6	-4.0 to -1.1	0.6 to 1.8	-10 to -1	
8	3.6 to 3.8	2.0 to 2.1	-3 to -3	
2	0.0 to 2.3	-0.2 to 1.5	-10 to 0	Threshold of 85 %
4	-4.0 to 2.8	-2.0 to 4.0	-10 to 0	
6	-4.0 to -0.8	0.1 to 2.3	-10 to -0	
8	2.2 to 4.0	-0.4 to 2.1	-10 to -3	

Similar to Table 1 in the main text, but with different threshold levels.

Supplementary Table 3: Overview of best fitting models for mode $_2S_{16}$ for variations in the depth range and position of the layer in which the scaling factors are varied.

Depth range of layer	Height above CMB	R_{LL}^{low}	R_{LL}	R_{SR}	Probability	L2-norm
2300km – 2891km	-	-	1.0	1.0	0.64	0.00511
2400km – 2891km	-	-	2.0	1.0	0.86	0.00331
2500km – 2891km	-	-	3.0	1.0	0.86	0.00339
2600km – 2891km	-	-	4.0	2.0	0.86	0.00245
2700km – 2891km	-	-	4.0	4.0	0.64	0.00729
2800km – 2891km	-	-	4.0	4.0	0.35	0.02358
2400km – 2791km	100km	0.3	2.0	1.0	0.86	0.00314
2400km – 2791km	100km	-1.0	2.0	1.0	0.86	0.00315
2400km – 2791km	100km	-2.0	2.0	1.0	0.86	0.00313
2400km – 2791km	100km	-4.0	1.0	1.0	0.64	0.00314
2300km – 2691km	200km	0.3	2.0	1.0	0.86	0.00337
2300km – 2691km	200km	-1.0	2.0	1.0	0.86	0.00374
2300km – 2691km	200km	-2.0	2.0	1.0	0.64	0.00385
2300km – 2691km	200km	-4.0	3.0	1.0	0.86	0.00461

Analysis based on Stoneley mode $_2S_{16}$ at structural degree s = 2.
R_{LL}^{low} indicates the scaling factor for the LLSVPs in the lowest 100 or 200 km of the mantle.

Supplementary Table 4: Overview of best fitting models for all Stoneley modes combined, similar to Supplementary Table 3.

Depth range of layer	Height above CMB	R_{LL}^{low}	R_{LL}	R_{SR}	Probability	L2-norm
2300km – 2891km	-	-	1.0	0.3	0.49	0.342
2400km – 2891km	-	-	1.0	0.3	0. 51	0.351
2500km – 2891km	-	-	1.0	1.0	0.47	0.405
2600km – 2891km	-	-	2.0	1.0	0.47	0.377
2700km – 2891km	-	-	2.0	2.0	0.47	0.382
2800km – 2891km	-	-	4.0	4.0	0.38	0.379
2400km – 2791km	100km	0.3	1.0	0.3	0.47	0.352
2400km – 2791km	100km	-1.0	1.0	0.3	0.44	0.354
2400km – 2791km	100km	-2.0	2.0	0.3	0.44	0.341
2400km – 2791km	100km	-4.0	2.0	0.3	0.47	0.342
2300km – 2691km	200km	0.3	1.0	1.0	0.49	0.435
2300km – 2691km	200km	-1.0	2.0	0.3	0.44	0.333
2300km – 2691km	200km	-2.0	3.0	0.3	0.42	0.326
2300km – 2691km	200km	-4.0	3.0	1.0	0.47	0.423

Analysis based on all Stoneley modes combined for structural degree s = 2.
R_{LL}^{low} indicates the scaling factor for the LLSVPs in the lowest 100 or 200 km of the mantle.

Supplementary Notes

Supplementary Note 1: Splitting function amplitudes

Splitting functions are measured using an iterated damped least squares inversion of free oscillation spectra. To robustly constrain the density scaling factor R in the lowermost mantle, it is crucial to have accurate estimates of seismic anomaly amplitudes, in addition to their shape [7]. Therefore, much care has been taken in the development of the splitting function data set, to ensure that the splitting function amplitudes are independent of the damping value of the inversion [8]. The norm damping value has been varied by several orders of magnitude and we have ensured to pick damping values for which the results do not change significantly when lowering the damping further.

Supplementary Note 2: Splitting function predictions for other Stoneley modes

Supplementary Fig. 4 shows observed and predicted splitting function maps for additional Stoneley modes, similar to Fig. 2 in the main text. Again, predictions for mantle model SP12RTS (Supplementary Fig. 4c, 4h and 4m) underestimate the splitting function amplitudes, which are reduced further by including dense LLSVPs (Supplementary Fig. 4d, 4i and 4n). A better fit to the observed splitting functions (Supplementary Fig. 4b, 4g and 4l) is only obtained for a positive value of R_{LL}, i.e. light LLSVPs (Supplementary Fig. 4e, 4j and 4o).

Supplementary Note 3: Effect of CMB topography on splitting function predictions

Supplementary Fig. 5 presents results of splitting function predictions for Stoneley mode ${}_2S_{16}$, now with CMB topography variations included in addition to density variations. Either models with dense LLSVPs and positive CMB topography scaling factors (Supplementary Fig. 5d, top), or models with light LLSVPs and negative CMB topography scaling factors (Supplementary Fig. 5f, bottom) improve the fit compared to the default density model (Supplementary Fig. 5e, centre). ${}_2S_{16}$ and other Stoneley modes, show a strong trade-off between the CMB topography and LLSVP density scaling factor, making it difficult to constrain both separately.

Supplementary Note 4: Best fitting models

Table 1 and Supplementary Table 2 present the ranges of resulting best fitting scaling factors for structural degrees $s > 2$, which fit the measurements within 95 (Table 1) and 90

or 85 % (Supplementary Table 2) of the maximum probability value. A possible lowermost mantle density model that would be compatible with current Stoneley mode splitting function data is shown in Supplementary Fig. 6. This density model shows ~0.9 % lower densities for the LLSVPs and ~1.1 % higher densities for the surrounding regions in the "Ring around the Pacific". It should be noted that this model is not necessarily unique as a range of models fit the splitting function data within their uncertainties, but all feature low density LLSVPs.

Supplementary Note 5: Comparison with the data of previous studies

Our analysis, suggesting light LLSVPs, contradicts previous normal mode density models that obtained higher densities for the LLSVPs [9–12]. However, these models were constructed with fewer data and without the new Stoneley mode measurements. It has been suggested that significant trade-offs between upper and lower mantle density structure existed in these studies and that a range of models with both positive and negative scaling factors fit the data equally well [13–15]. At the same time, it was suggested that reliable density models may be obtained in future when more normal mode data are available. We repeat our two-parameter model space search for the subset of modes available in the HT96+RR98 data set (Supplementary Table 1) using both the original and updated splitting measurements, reported in Supplementary Fig. 7. For the original HT96+RR98 measurements [5-6], we indeed find best fitting models with negative LLSVP density scaling factors, i.e. R_{LL} = −0.7 to −1.3 (Supplementary Fig. 7b). Using updated measurements for these modes from the DRH13 data set, we also find negative scaling factors for the LLSVPs: R_{LL} = −2.4 to −3.1 (Supplementary Fig. 7c). On the other hand, the Stoneley modes (Fig. 3c in the main text) find positive scaling factors for the LLSVPs with values between 1.7 and 1.9. In contrast, a model space search using all available modes of the DRH13+KDR13 data set [3-4] (Supplementary Fig. 7a) produces a very broad region of best fitting models, with both positive and negative values of R_{LL}. In the three-parameter model space search, where we include CMB topography variations (Supplementary Fig. 7d–f), we observe similar patterns as in Supplementary Fig. 7a–c. For every value of H, both positive and negative values of R_{LL} are found for all modes of the DRH13+KDR13 data sets (Supplementary Fig. 7d. The original measurements of the HT96+RR98 data sets (Supplementary Fig. 7e) also have difficulty resolving the sign of R_{LL} for different values of H. However, the updated measurements for the HT96+RR98 data sets (Supplementary Fig. 7f) show a similar pattern to the Stoneley

modes (Fig. 3f in the main text), except that the shift to the different model class happens for negative values of H already, resulting in dynamically feasible models with dense LLSVPs.

We believe that by focusing on the Stoneley modes, we are able to extract the signal originating from the deep mantle. This philosophy is similar to the approach in body wave studies of using core-diffracted and reflected waves and not upper mantle phases to study the CMB region. Supplementary Fig. 7 also illustrates that using the HT96+RR98 selection of normal modes masks the signal due to the lowermost mantle, as the variations in probability values are very small (Supplementary Fig. 7b and c). In addition, none of the models in the entire model space search pass our observability criterion [16] due to the large uncertainties in the original splitting function measurements. Thus, we confirm the previous findings of dense LLSVPs using the HT96+RR98 data set. However, we also agree with subsequent studies that these data were insufficient to constrain the density structure of the lowermost mantle robustly. In contrary, we state that our Stoneley mode splitting data with unique sensitivity to the lowermost mantle are best fitted by light LLSVPs.

Supplementary Note 6: Fit to other lower mantle sensitive modes

To investigate whether light LLSVPs (as preferred by the Stoneley modes) are incompatible with the splitting functions of other modes sensitive to the lowermost mantle, we examine the fit of these modes to the best fitting density models listed in Supplementary Table 2. We define a mode to be significantly sensitive to the lowermost mantle if the sensitivity to structure below 2500 km depth is at least half the maximum sensitivity in the mantle. Supplementary Fig. 8 shows the probability values and L2-norm changes with respect to the fit for SP12RTS for the resulting 26 modes. Most non-Stoneley modes sensitive to the lower mantle show small changes in the L2-norm (Supplementary Fig. 8c-d), with only modes ${}_0S_2$, ${}_0S_7$ and ${}_{14}S_9$ strongly preferring denser LLSVPs (dashed lines). However, the Stoneley modes have much higher L2-norm values for dense LLSVPs (Supplementary Fig. 8d), whereas they decrease very pronouncedly for light LLSVPs (Supplementary Fig. 8c). This discrepancy is likely to be due to unmodelled structure in the mid mantle. Other lower mantle sensitive modes have broader sensitivity kernels in the lower mantle and are therefore affected by structure in the mid mantle that is unaccounted for. On the other hand, the Stoneley modes are limited in sensitivity to depths near the CMB and will not be affected by unmodelled structures at mid mantle depths. Additionally, our inferences of light LLSVPs are based on

the range of possible models that fit the splitting functions within their uncertainties, not on an L2-norm. Inspection of the probability values of these 26 modes indicates that modes such as ${}_0S_2$ (which has been used to infer dense LLSVPs in recent studies [17]) cannot be used to distinguish between light and dense LLSVPs, as the probability values are the same. ${}_0S_2$ is the longest period normal mode, and consequently very difficult to measure, with only 78 usable spectra (8 earthquakes) available for the splitting function measurement [18]. The associated uncertainties in the splitting function coefficients are therefore very large, and almost any density model can be fitted within these uncertainties. As comparison, Stoneley modes are typically measured using 2000–3000 spectra from 90 earthquakes, giving rise to much smaller uncertainties [3]. Furthermore, the average probability of these 26 modes is higher for models with light LLSVPs (0.38 and 0.36 in Supplementary Fig. 8a) than for models with dense LLSVPs (0.25 and 0.25 in Supplementary Fig. 8b) or for SP12RTS (0.26).

Supplementary Note 7: Effect of default density scaling factor

The inversion for shear- and compressional-wave velocity variations in SP12RTS assumes R = 0.3 [1]. Therefore, it is possible that the results of our analysis are dependent on the value of R in SP12RTS. To test this, we make use of a different version of SP12RTS where R = 0.0 was used, thus not accounting for any density variations in the mantle. The resulting density models are very similar as evidenced in Supplementary Fig. 9, with slightly smaller amplitudes for R = 0.3 in SP12RTS (Supplementary Fig. 9a). With these models, we repeat our model space search for R_{LL}, R_{SR} and H. Despite the small differences in the density input models, the resulting probability plots for the three different cases are very similar. We systematically find positive values of R_{LL} and R_{SR}, even when no density variations are included anywhere else in the mantle (Supplementary Fig. 9b).

Supplementary Note 8: Testing trade-offs with shear-wave velocity structure

To test possible trade-offs between velocity and density structure for the Stoneley modes, we have repeated our model space search for two different models of velocity structure. Model SP12RTS_low_D is a lower damped version of SP12RTS and hence features larger velocity amplitudes, about 1.4 times as large as in SP12RTS (Supplementary Fig. 10). This model allows us to investigate whether dense LLSVPs can be obtained when the shear-wave velocity amplitudes are larger, as the sensitivity kernels of the Stoneley modes for shear-wave velocity and density are similar in the lowermost mantle (Supplementary Fig. 3).

Supplementary Fig. 11 to 13 are equivalent to Fig. 2 to 4 in the main text, but we now use SP12RTS_low_D to describe the velocity structure in the entire mantle. The predicted splitting function maps (compare Supplementary Fig. 11 and Fig. 2 in the main text) are very similar, albeit that the amplitudes are slightly larger when using model SP12RTS_low_D. The results of the model space search without CMB topography variations (compare Supplementary Fig. 12a-b and Fig. 3a-c in the main text) also show that the Stoneley mode splitting functions are fitted best by density models with positive values of R_{LL} in both cases (R_{LL} = 1.5 and R_{SR} = −0.9 for SP12RTS_low_D versus R_{LL} = +1.7 to +1.9 and R_{SR} = +0.4 to +0.9 for SP12RTS). The main difference is that similar fits are obtained for more negative values of R_{SR}. At the same time, we observe that the other mode selections (compare Supplementary Fig. 12c-d with Supplementary Fig. 7a-c) are not as sensitive to the density structure of the lower mantle, but that the probability values are larger than when we use SP12RTS. The fact that such alternative models of mid mantle velocity structure increase the probability values of these modes likely indicates that the discrepancy between the Stoneley modes and these modes is due to unmodelled structure at these depths. When CMB topography variations are included (compare Supplementary Fig. 12e-h with a combination of Fig. 3d-f in the main text and Supplementary Fig. 7d-f), we find similar patterns as before. The Stoneley modes find positive values of R_{LL} for $H < 0$, whereas both positive and negative values of R_{LL} fit the KDR13+DRH13 and HT96+RR98 mode selections equally well. The amplitude of R_{LL} we find for the best fitting model depends on the CMB topography scaling factor (Supplementary Fig. 13). In this case, the best fitting model for the Stoneley modes is characterised by R_{LL} = 1.5, R_{SR} = −0.9 and H = 0.

Supplementary Note 9: Testing trade-offs with compressional-wave velocity structure

Additionally, we investigate the effects of the compressional-wave velocity structure by using model SP12RTS_P_scaled, in which we have scaled the P-wave velocity variations to shear-wave velocity variations through a depth-dependent scaling of 0.5 at the Earth's surface and 0.333 at the CMB (as assumed in the S40RTS model [19]). Maps of dlnV_P are consequently scaled versions of dlnV_S and the relative amplitudes of the shear-wave and compressional-wave velocity variations are substantially different.

In Supplementary Fig. 15 to 18 we show the same results while using SP12RTS_P_scaled instead of SP12RTS. The splitting function maps (Supplementary Fig. 14) now show slightly

lower amplitudes, but the differences with SP12RTS are smaller than when the shear-wave velocity structure was changed (Supplementary Fig. 11). Supplementary Fig. 15 (compare to Fig. 3 in the main text), which presents the results of the model space search for the different mode selections, illustrates this further. The Stoneley modes are again fitted best for positive values of R_{LL} (R_{LL} = 1.2 and R_{SR} = 1.0 for SP12RTS_P_scaled). We also note that for all panels the probability values are lower, indicating that we do not fit the splitting function data as well as when we use SP12RTS. This illustrates that the P-wave velocity structure in SP12RTS is strongly preferred by the normal modes. Again, when CMB topography variations are included, we obtain similar patterns as before (Supplementary Fig. 15e-h and Supplementary Fig. 16), indicating that the same two model classes arise. The region of best fitting models remains substantially larger for other mode selections than for the Stoneley modes together. Nevertheless, the best fitting models are still characterised by positive density scaling factors with R_{LL} = 0.4, R_{SR} = 0.6 and H = −7.

Supplementary Note 10: Velocity-density trade-offs

As Supplementary Fig. 11 to 16 demonstrate, we consistently find positive values of R_{LL} with the amplitude depending primarily on the CMB topography scaling factor. Using model SP12RTS_low_D has a larger effect than using model SP12RTS_P_scaled, confirming the notion that the Stoneley modes respond more to variations in shear-wave velocity than P-wave velocity. Naturally, a full model space search of both velocity and density with all uncertainties taken into account is preferable over these experiments. However, they indicate that low densities are strongly preferred by the Stoneley mode data for reasonable models of lowermost mantle velocity structure.

Supplementary Note 11: Presence of a lower dense layer

We have fixed the depth, below which we vary the density scaling factors, to 2500 km depth, corresponding approximately to the anomalous region found in recent lower mantle tomographic clustering analyses [9]. Similar density models are obtained for mode ${}_2S_{16}$ when we change this depth (R_{LL} between 3 and 4 and R_{SR} between 1 and 2), with the best fit to the splitting function measurements found for depths between 2400 and 2600 km (Supplementary Table 3). At the same time, the L2-norm only degrades substantially for depths above 2400 km or below 2600 km. Therefore, an intermediate depth of 2500 km has generally been assumed in our model space search. Similar results are obtained when we

consider all Stoneley modes together (Supplementary Table 4). A greater depth requires a larger value of R_{LL} and R_{SR}, indicating that the modes are primarily sensitive to the total amount of heterogeneity in the lowermost mantle. If we allow the LLSVPs (of the same thickness) to be raised above the CMB and include an additional scaling factor $R_{\mathrm{LL}}^{\mathrm{low}}$in the mantle below this, we find very similar probability values for both positive and negative values of $R_{\mathrm{LL}}^{\mathrm{low}}$ for Stoneley mode ${}_2S_{16}$ (Supplementary Table 3). For LLSVPs raised 100 km above the CMB, the L2-norm is practically the same, whereas the norm increases for LLSVPs raised 200 km above the CMB. This suggests that even though the Stoneley modes have superior sensitivity to density structure, it is difficult to constrain the value of R_{LL} in the lowest 100 km of the mantle. For all Stoneley modes combined (Supplementary Table 4), probability values are generally lower when we allow for a dense layer just on top of the CMB. In addition, larger positive values of R_{LL} are required to compensate for this lower dense layer, implying that the Stoneley modes prefer an overall, lighter depth-average for the LLSVPs.

Supplementary References

1. Koelemeijer, P., Ritsema, J., Deuss, A. & van Heijst, H.-J. SP12RTS: a degree-12 model of shear- and compressional-wave velocity for Earth's mantle. *Geophys. J. Int.*, **204**, 1024–1039 (2016).
2. Mooney, W., Laske, G. & Masters, T. CRUST 5.1: A global crustal model at 5× 5 ◦. *J. Geophys. Res.* **103**, 727–747 (1998).
3. Koelemeijer, P., Deuss, A. & Ritsema, J. Observations of core-mantle boundary Stoneley modes. *Geophys. Res. Lett.* **40**, 2557–2561 (2013).
4. Deuss, A., Ritsema, J. & Van Heijst, H.-J. A new catalogue of normal-mode splitting function measurements up to 10 mHz. *Geophys. J. Int.,* **192**, 920-937 (2013).
5. He, X. & Tromp, J. Normal-mode constraints on the structure of the Earth. *J. Geophys. Res.* **101**, 20–53 (1996).
6. Resovsky, J. S. & Ritzwoller, M. H. New and refined constraints on three-dimensional Earth structure from normal modes below 3 mHz. *J. Geophys. Res.* **103**, 783–810 (1998).
7. Masters, G., Laske, G., Bolton, H. & Dziewonski, A. The relative behavior of shear velocity, bulk sound speed, and compressional velocity in the mantle: Implications for chemical and thermal structure. *Geophys. Monograph AGU*, **117**, 63–87 (2000).

8. Koelemeijer, P. J. Normal mode studies of long wavelength structures in Earth's lowermost mantle. *PhD thesis, University of Cambridge* (2014).
9. Ishii, M. & Tromp, J. Normal-mode and free-air gravity constraints on lateral variations in velocity and density of Earth's mantle. *Science* **285**, 1231–1236 (1999).
10. Ishii, M. & Tromp, J. Constraining large-scale mantle heterogeneity using mantle and innercore sensitive normal modes. *Phys. Earth Planet. Inter.* **146**, 113–124 (2004).
11. Trampert, J., Deschamps, F., Resovsky, J.&Yuen, D. Probabilistic tomographymaps chemical heterogeneities throughout the lower mantle. *Science* **306**, 853–856 (2004).
12. Mosca, I., Cobden, L., Deuss, A., Ritsema, J. & Trampert, J. Seismic and mineralogical structures of the lower mantle from probabilistic tomography. *J. Geophys. Res*. **117**, B06304 (2012).
13. Resovsky, J. & Ritzwoller, M. Regularization uncertainty in density models estimated from normal mode data. *Geophys. Res. Lett.* **26**, 2319–2322 (1999).
14. Masters, G., Laske, G. & Gilbert, F. Matrix autoregressive analysis of free-oscillation coupling and splitting. *Geophys. J. Int.* **143**, 478–489 (2000).
15. Romanowicz, B. Can we resolve 3D density heterogeneity in the lower mantle? *Geophys. Res. Lett*. **28**, 1107–1110 (2001).
16. Koelemeijer, P., Deuss, A. & Trampert, J. Normal mode sensitivity to Earth's D" layer and topography on the core–mantle boundary: what we can and cannot see. *Geophys. J. Int.* **190**, 553–568 (2012).
17. Moulik, P. & Ekström, G. The relationships between large-scale variations in shear velocity, density, and compressional velocity in the Earth's mantle. J. Geophys. Res. 121, 2737–2771 (2016).
18. Deuss, A., Ritsema, J. & van Heijst, H.-J. Splitting function measurements for Earth's longest period normal modes using recent large earthquakes. *Geophys. Res. Lett.* **38**, L04303 (2011).
19. Ritsema, J., Deuss, A., van Heijst, H.-J. & Woodhouse, J. H. S40RTS: a degree-40 shear-velocity model for the mantle from new Rayleigh wave dispersion, teleseismic traveltime and normal-mode splitting function measurements. *Geophys. J. Int.* **184**, 1223–1236 (2011).
20. Lekic, V., Cottaar, S., Dziewonski, A. & Romanowicz, B. Cluster analysis of global lower mantle tomography: A new class of structure and implications for chemical heterogeneity. *Earth Planet. Sci. Lett.* **357**, 68–77 (2012).
